# Origin, loss, and regain of self-incompatibility in angiosperms

**DOI:** 10.1093/plcell/koab266

**Published:** 2021-11-04

**Authors:** Hong Zhao, Yue Zhang, Hui Zhang, Yanzhai Song, Fei Zhao, Yu’e Zhang, Sihui Zhu, Hongkui Zhang, Zhendiao Zhou, Han Guo, Miaomiao Li, Junhui Li, Qiang Gao, Qianqian Han, Huaqiu Huang, Lucy Copsey, Qun Li, Hua Chen, Enrico Coen, Yijing Zhang, Yongbiao Xue

**Affiliations:** 1 State Key Laboratory of Plant Cell and Chromosome Engineering, Institute of Genetics and Developmental Biology, and the Innovation Academy of Seed Design, Chinese Academy of Sciences, Beijing 100101, China; 2 University of Chinese Academy of Sciences, Beijing 100049, China; 3 College of Life Science, Northwest Normal University, Lanzhou 730070, China; 4 National Key Laboratory of Plant Molecular Genetics, CAS Center for Excellence in Molecular Plant Sciences, Shanghai Institute of Plant Physiology and Ecology, Shanghai Institutes for Biological Sciences, Chinese Academy of Sciences, Shanghai 200032, China; 5 Beijing Institute of Genomics, Chinese Academy of Sciences, and China National Centre for Bioinformation, Beijing 100101, China; 6 John Innes Centre, Norwich NR47UH, UK; 7 State Key Laboratory of Genetic Engineering, Collaborative Innovation Center of Genetics and Development, Department of Biochemistry, Institute of Plant Biology, School of Life Sciences, Fudan University, Shanghai 200438, China; 8 Jiangsu Co-Innovation Center for Modern Production Technology of Grain Crops, Yangzhou University, Yangzhou 225009, China

## Abstract

The self-incompatibility (SI) system with the broadest taxonomic distribution in angiosperms is based on multiple *S*-*locus F*-*box* genes (*SLF*s) tightly linked to an *S*-*RNase* termed type-1. Multiple SLFs collaborate to detoxify nonself S-RNases while being unable to detoxify self S-RNases. However, it is unclear how such a system evolved, because in an ancestral system with a single SLF, many nonself S-RNases would not be detoxified, giving low cross-fertilization rates. In addition, how the system has been maintained in the face of whole-genome duplications (WGDs) or lost in other lineages remains unclear. Here we show that SLFs from a broad range of species can detoxify S-RNases from *Petunia* with a high detoxification probability, suggestive of an ancestral feature enabling cross-fertilization and subsequently modified as additional SLFs evolved. We further show, based on its genomic signatures, that type-1 was likely maintained in many lineages, despite WGD, through deletion of duplicate *S-*loci. In other lineages, SI was lost either through *S-*locus deletions or by retaining duplications. Two deletion lineages regained SI through type-2 (Brassicaceae) or type-4 (Primulaceae), and one duplication lineage through type-3 (Papaveraceae) mechanisms. Thus, our results reveal a highly dynamic process behind the origin, maintenance, loss, and regain of SI.

## Teaser

Genetic, functional, phylogenomic, and ancestral state reconstruction studies reveal a highly dynamic process behind the origin, maintenance, loss, and regain of self-incompatibility in angiosperms.

##  

**Figure:**
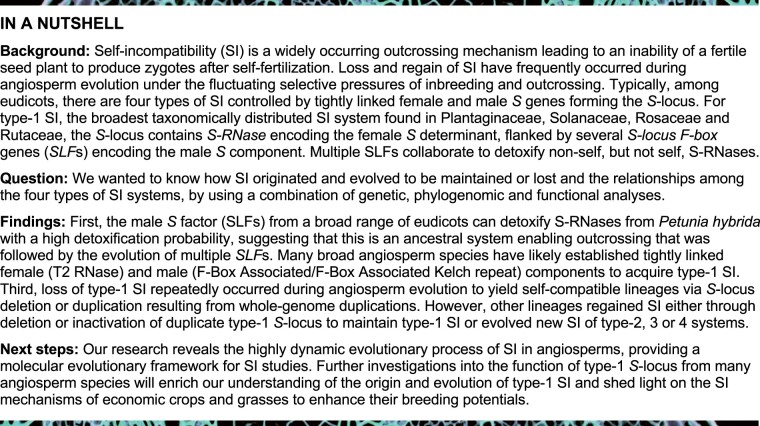


## Introduction

Self-incompatibility (SI), the inability of a fertile seed plant to produce a zygote after self-pollination, serves as a widely occurring outcrossing mechanism to prevent inbreeding in angiosperms. Approximately 40% of all angiosperm species possess SI. Various SI systems have evolved and are classified based on their associations with floral morphology (homo or heteromorphic SI; heterostyly), the genetic control of pollen SI phenotypes (sporophytic SI [SSI] or gametophytic SI [GSI]) or the number of *S*-loci (single to multiple) (de Nettancourt, 2001; Takayama and Isogai, 2005; Franklin-Tong, 2008; Zhang et al., 2009; Fujii et al., 2016). SI has been frequently lost and regained during evolution because of fluctuating selective pressures on selfing and outcrossing (de Nettancourt, 2001; Franklin-Tong, 2008). However, the mechanisms underlying origins and losses remain unclear. Here we address this problem through a combination of phylogenetic and functional tests across a broad range of taxa.

The system with the broadest taxonomic distribution, which we term type-1 SI, is gametophytic and based on linked pistil and pollen *S* components, corresponding to *S*-*RNase* and *S*-*locus F*-*box* (*SLF*), also named *S*-*haplotype-specific F*-*box* (*SFB*), respectively. So far, type-1 SI has been found in four eudicot families: Solanaceae, Plantaginaceae, Rosaceae, and Rutaceae, spanning two major clades (superrosids and superasterids; Anderson et al., 1986; McClure et al., 1989; Sassa et al., 1996; Xue et al., 1996; Lai et al., 2002; Ushijima et al., 2003; Sijacic et al., 2004; Qiao et al., 2004b; Sassa et al., 2007; Liang et al., 2020). In each case, an exemplar species (*Petunia inflata*, Spanish snapdragon [*Antirrhinum hispanicum*], wild cherry [*Prunus avium*], and pomelo [*Citrus maxima*]) has been shown to carry the *S*-locus containing an *S-RNase* flanked by a cluster of 9–37 *SLF* genes (Williams et al., 2014a; Kubo et al., 2015; Li et al., 2019; Liang et al., 2020). These genes function in SI, based on their specific expression in pistil and pollen, multiple alleles, correlation with *S* genotypes, mutations, and gene transformations that alter incompatibility or compatibility (Lee et al., 1994; Murfett et al., 1994; Royo et al., 1994; Sassa et al., 1997; Lai et al., 2002; Sijacic et al., 2004; Ushijima et al., 2004; Qiao et al., 2004b; Xue et al., 2009; Ye et al., 2018; Liang et al., 2020). Phylogenetic trees of T2 *RNase* genes have shown that *S-RNase*s from species of both superrosids and superasterids cluster in a monophyletic group, suggesting a single origin of type-1 SI in the core eudicots (Xue et al., 1996; Igic and Kohn, 2001; Steinbachs and Holsinger, 2002; Vieira et al., 2008; Ramanauskas and Igic, 2017; Liang et al., 2020). Consistent with this idea, eight T2 RNases identified in monocots species such as rice (*Oryza sativa*) group into two other classes of T2 RNases (Classes I and II) compared to S-RNases (Class III) (MacIntosh et al., 2010). However, a ribonuclease T2 family member (Aco001100) from pineapple (*Ananas comosus*) was shown to be tightly linked to several genes encoding F-box family members (Chen et al., 2019), indicating a likely presence of type-1 SI in monocots, although this remains to be confirmed through genetic linkage studies.

The type-1 SI system operates through multiple pollen-specific SLFs from one haplotype detoxifying pistil-specific S-RNases from other haplotypes, while not detoxifying S-RNases from its own haplotype (Sijacic et al., 2004; Qiao et al., 2004a, 2004b; Kubo et al., 2010; Liu et al., 2014; Zhao et al., 2021). Transgenic studies show that a single SLF can detoxify about 50% S-RNases from the same species (detoxification probability of 0.5), so multiple SLFs are needed within each haplotype to ensure high levels of cross-compatibility (Kubo et al., 2010; Williams et al., 2014b). However, it is unclear how such a system originated. If the ancestral *S-*locus contained a single *SLF* linked to an *S-RNase* (Sakai and Haluka, 2014; Sakai, 2016), a detoxification probability of 0.5 would lead to each haplotype only being able to pollinate 25% of females (assuming females are heterozygous and thus carry two S-RNases that need to be detoxified).

Type I SI system has been lost in multiple lineages (Fujii et al., 2016). Such losses may arise through several routes: (1) duplication of the *S-*locus to create two recombining haplotypes within the same genome, allowing SLFs from one haplotype to detoxify S-RNases from the other; (2) inactivation of the *S-RNase*; and (3) deletion of the entire *S-*locus. However, the relative contribution of these three mechanisms is not known. Moreover, it is unclear how type-1 SI was maintained in the face of whole-genome duplications (WGDs), which would have caused breakdown of SI via Route 1.

Depending on the loss mechanism, SI may have been regained through deletion of duplicate *S*-loci, reactivation of an *S*-*RNase*, or evolution of a new SI system. The latter process likely accounts for the other types of SI in eudicots (Fujii et al., 2016). Type-2 SI is the sporophytic Brassicaceae-type SI, controlled by a male *S*-locus cysteine-rich (SCR) protein/*S*-locus protein 11 and a female *S*-locus receptor kinase (SRK; Schopfer et al., 1999; Suzuki et al., 1999; Takasaki et al., 2000; Takayama et al., 2000). Type-3 is the gametophytic Papaveraceae-type SI, possessing the common poppy (*Papaver rhoeas*) stigma *S* (PrsS) and *P*. *rhoeas* pollen *S* (PrpS) (Foote et al., 1994; Wheeler et al., 2009). Type 4 is the sporophytic heterostyly of *Primula*, involving the *S*-locus supergene consisting of five genes encoding style length-determining cytochrome P450 (CYP), anther position-controlling GLOBOSA (GLO), a functionally unknown Conserved Cysteine Motif (CCM), Pumilio-like RNA-binding protein (PUM) and a Kelch repeat F-Box (KFB; Huu et al., 2016, 2020; Li et al., 2016). However, it is unclear what caused inactivation of the type-1 *S*-loci in these cases where SI was regained by a new mechanism.

Here we use a combination of phylogenetic and functional approaches to reveal a highly dynamic picture of SI evolution. We show that SLFs from a broad range of species can detoxify S-RNases from *Petunia* with a high detoxification probability, suggesting a likely mechanism for how type-1 SI might have originated. We further show, based on the distribution of type-1 *S*-locus signatures in the genome, that a type-1 SI system was maintained in many lineages, despite WGD, through deletion of duplicate *S-*loci. In other lineages, SI was lost either through deletion or duplications of the *S-*locus. Inactivation of the S-RNase was only detected in horticulturally selected lines, suggesting this route to self-compatibility (SC) is not favored in natural populations. Two deletion lineages regained SI through type-2 or type-4 mechanisms, while one duplication lineage regained SI through a type-3 mechanism.

## Results

### Phylogenetic analyses reveal that type-1 SI traces back to a single origin

**Figure 1:**
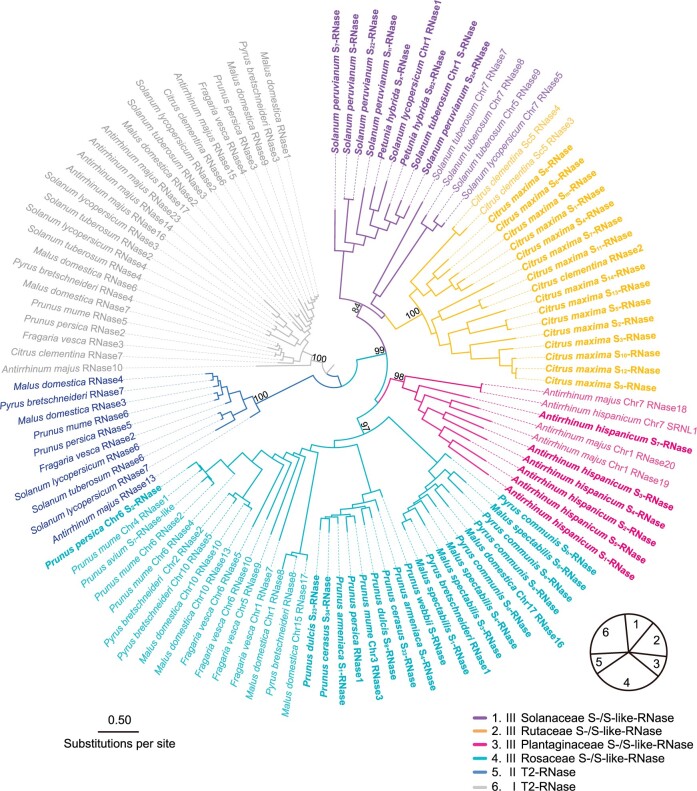
ML tree of the T2 RNase superfamily of the four exemplar families. ML phylogenetic tree of T2 RNases from 12 species of the four exemplar families with bootstrap confidence values >50%. I, II, and III indicate Class I, II, and III T2 RNases. Class III T2 RNases from different families and other types of T2 RNases are indicated by different branch colors. Bold fonts indicate functionally defined S-RNases. Chr, Chromosome; Sc, Scaffold. Please refer to Supplemental File S2 for the detailed bootstrap values.

Type-1 SI involves pistil S-RNases, which are members of the T2-type RNases. To elucidate the evolution of type-1 SI, we performed phylogenetic analyses of all T2 RNases from 12 species in four eudicot families (Plantaginaceae, Solanaceae, Rosaceae, and Rutaceae), two from monocot families (Poaceae and Bromeliaceae) and other angiosperms as well as gymnosperm species (Supplemental Data Set S1). Their T2 RNases fell into three clades: Classes I, II, and III. All S-RNases from the eudicot exemplar species were in Class III T2 RNase clade (Figure  1;Supplemental Figure S1). In addition to S-RNases, Class III T2 RNases of eudicot exemplar species included paralogs separate from the *S*-locus. For example, *Antirrhinum* contained a Class III T2 RNase on chromosome 7, whereas its *S*-locus maps to chromosome 8 (Li et al., 2019). These paralogs typically belonged to the same clade as *S-RNase*s from the same plant family, suggesting that they arose through duplications within the family lineage. We refer to such paralogs as *S*-*like*-*RNase*s.

**Figure 2:**
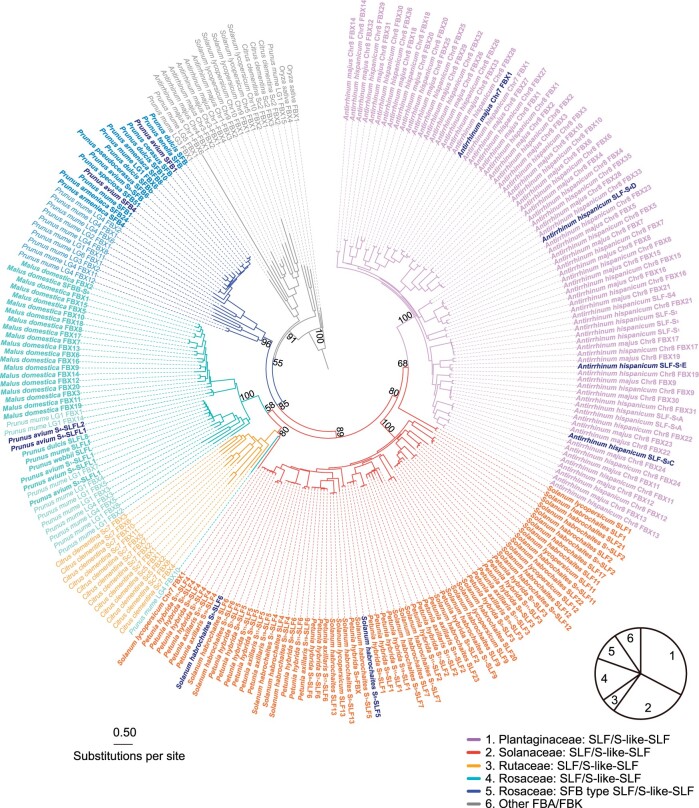
ML tree of FBA/FBKs of the four exemplar families. ML phylogenetic tree of FBAs/FBKs from 12 species of the four exemplar families with those from rice as a root and bootstrap confidence values > 50%. The SLFs from different families are indicated by different branch colors. Bold fonts indicate functionally defined SLFs. Green bold fonts indicate SLFs used for functional analysis. Please refer to Supplemental File S2 for the detailed bootstrap values.

To explore the evolution of the pollen component, we also performed phylogenetic analysis of SLF and other F-Box Associated/F-Box Associated Kelch (FBA/FBK) repeat proteins encoded by those genes linked to *S*-*like*-*RNase*s in the eudicot exemplar species, or to Class I/II T2 *RNase*s of monocot and other angiosperm species (Supplemental Data Set S2). The FBA/FBKs for the eudicot exemplar species all belonged to a single clade, which we refer to as the *SLF* subfamily, while those from monocots and other angiosperm species clustered into an outgroup (Figure  2;Supplemental Figure S2), thus supporting a single origin.

**Figure 3:**
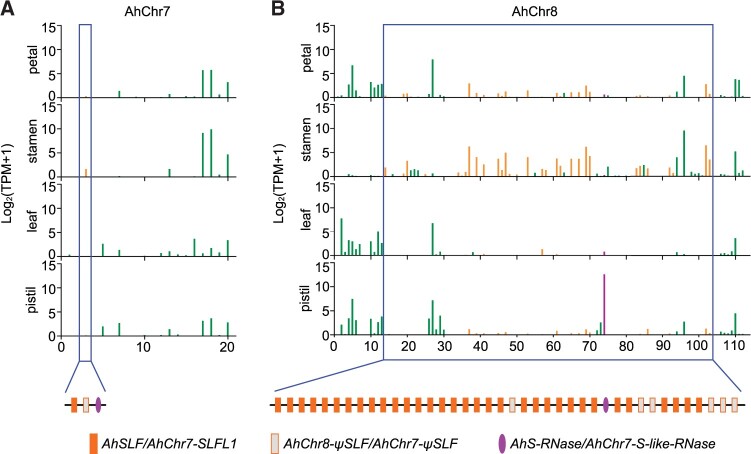
Transcriptional profiles of the type-1 *S-* and *S*-like-locus of *A. hispanicum*. The blue boxed regions denote the type-1 *S*-like-locus (A) and *S-*locus (B) covering 33 kb and 1.23 Mb, respectively. *x*-axes represent gene numbers. The expression levels of the *AhSLF* and *AhChr7-SLFL1* genes are shown in orange, *AhS-RNase*/*AhChr7-S-like-RNase* in violet and other unrelated genes in the region as green rectangles, for the indicated tissues on the *y*-axes. The violet ovals indicate the *AhS-RNase*/*AhChr7-S-like-RNase* and the rectangles the *AhSLF*s/*AhChr7-SLFL1* (orange) or *ψSLF* (light gray).

As with *S-RNase*s, the *SLF* subfamily included paralogs outside the *S*-locus in exemplar species. These members typically belonged to the same clade as the *SLF*s from the same plant family, suggesting that they arose through duplications within the family lineage (Figure  2). We refer to such members as *S-like SLF*s. In exemplar species, they were always closely linked to *S*-*like*-*RNase*s. For example, *Antirrhinum* contained an *S-like SLF* (*AhChr7-SLFL1* and *AmChr7-SLFL1*) closely linked to an *S-like-RNase* on chromosome 7, suggesting duplication of an ancestral *S*-locus region (Li et al., 2019). However, in contrast to the *S-*locus, which contained multiple *SLF*s, the duplicated *S-*like locus on chromosome 7 contained only two *S-like SLF*s, one of which was a pseudogene (*AhChr7-ψSLF*) that was not expressed (Figure  3).

### *SLF*s and *S-like SLF*s can detoxify *S-RNase*s across superasterids

Expression of an *SLF* from a different *S*-haplotype in pollen will protect the pollen from its own encoded S-RNase, leading to breakdown of incompatibility, termed competitive interaction (Crane and Lewis, 1942; Lewis, 1947; Sijacic et al., 2004; Qiao et al., 2004b; Kubo et al., 2010; Zhao et al., 2021). These assays have largely been carried out using *SLF*s from the same species as the target *S-RNase.*

To examine the detoxification potential of *SLF*s and *S-like SLF*s across taxa, we introduced two *SLF*s from the *S_5_* locus of wild tomato (*Solanum habrochaites*) (*ShS_5_-SLF5* or *ShS_5_-SLF6*) (Supplemental Data Set S3) under the control of strong pollen promoters into the self-incompatible *Petunia hybrida* genotype *S_3_S_3L_* (*PhS_3_S_3L_*) (Supplemental Figure S3A). The transgenic plants *ShS_5_-SLF5 PhS_3_S_3L_* and *ShS_5_-SLF6 PhS_3_S_3L_* gained SC (Supplemental Figure S3B; Supplemental Table 1). Self-progeny all carried transgenes, indicating that the observed SC results from competitive interaction (Supplemental Figure S3C). *S*-haplotype determinations showed that the transgenic line *ShS_5_-SLF5 PhS_3_S_3L_* #2 produces 12 *S_3_S_3L_* and nine *S_3L_S_3L_* progeny, but no *S_3_S_3_* progeny. Similarly, the transgenic line *ShS_5_-SLF5 PhS_3_S_3L_* #5 produced 14 *S_3_S_3L_* and nine *S_3L_S_3L_* progeny, but no *S_3_S_3_* progeny, indicating that ShS_5_-SLF5 can inactivate the *P.* *hybrida* S_3L_-RNase but not the S_3_-RNase (Supplemental Table S1). The transgenic line *ShS_5_-SLF6 PhS_3_S_3L_* #1 produced 11 *S_3_S_3_* and 12 *S_3_S_3L_* progeny, and no *S_3L_S_3L_* progeny, while the transgenic line *ShS_5_-SLF6 PhS_3_S_3L_* #3 produced 13 *S_3_S_3_* and 10 *S_3_S_3L_* progeny and no *S_3L_S_3L_*. Thus, ShS_5_-SLF6 was able to inactivate the PhS_3_-RNase but not the PhS_3L_-RNase (Supplemental Table S1).

To examine the function of *SLF*s across a broader taxonomic distance, we introduced three *Antirrhinum SLF*s, one *S-like SLF* (Supplemental Data Set S3), and the FBA/FBK-encoding gene from *Antirrhinum majus Chr1-FBX6* (*AmChr1-FBX6*) that does not belong to the *SLF* subfamily, under the control of strong pollen promoters into the *PhS_3_S_3L_* background. Aniline blue staining showed that SI breaks down in transgenic plants expressing the *SLF*s and *S-like SLF* genes but not for the FBA/FBK-encoding gene (Supplemental Figure S4, A and B). Polymerase chain reaction (PCR) analysis showed that all self-progeny carry transgenes (Supplemental Figure S4C). For the transgenic lines carrying *Antirrhinum SLF*s, progeny testing showed that *Antirrhinum* SLFs can inactivate both PhS_3L_- and PhS_3_-RNase (Supplemental Table S2).

Expression of the *S-like SLF* (*AmChr7-SLFL1*) caused breakdown of SI by inactivating the PhS_3L_-RNase (Supplemental Table S2). This result raised the question as to why the *S-like SLF* did not cause breakdown of SI in *A.* *hispanicum* by inactivating its S-RNase*.* Transcriptome analysis showed that *AhChr7-SLFL1* is expressed at a low level in the stamens of *A.* *hispanicum* compared to *AhChr7-ψSLF* and *S-*locus *SLF*s, 10–12 of which were expressed at high levels (Figure  3;Supplemental Data Sets S4 and S5). Thus, low expression of *AhChr7-SLFL1* may prevent it from causing breakdown of SI within *A. hispanicum.* In addition, the *S-like*-*RNase* was expressed at low levels in styles relative to the *S-RNase*, suggesting that it does not contribute to SI (Figure  3).

In summary, the ability to inactivate S-RNases is a feature of the *SLF* subfamily and acts across the Plantaginaceae and Solanaceae (superasterids).

### *SLF*s and *S-like SLF*s can detoxify *S-RNase*s across the Solanaceae and Rosaceae

**Figure 4:**
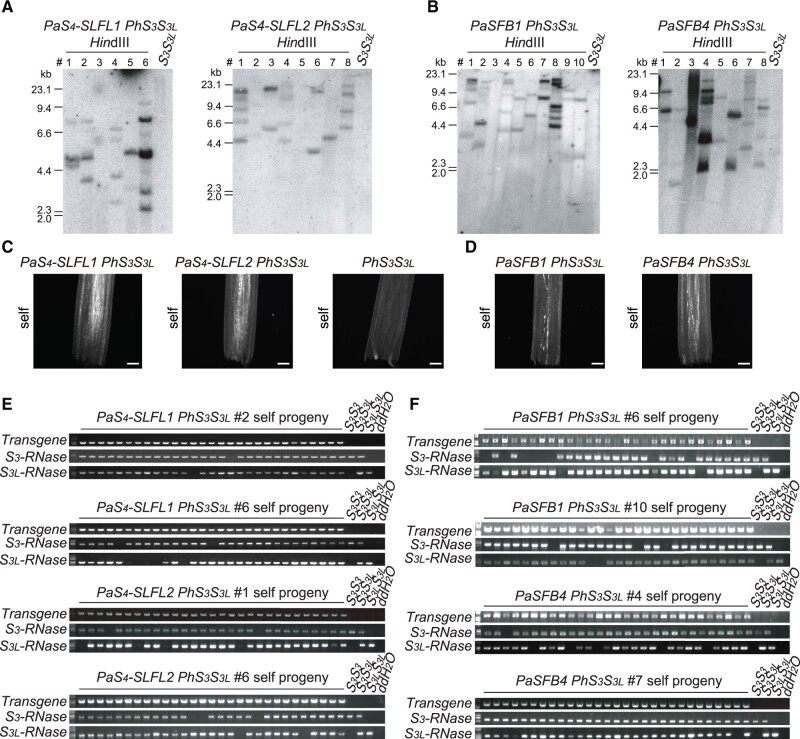
Both *SLF* and *SFB* type *SLF*s of *P. avium* function as pollen *S* factors. A and B, Southern blot analysis of T_0_ transgenic lines harboring *PaS_4_-SLFL1*, *PaS_4_-SLFL2*, *PaSFB1*, and *PaSFB4*. C and D, Aniline blue staining of self-pollen tubes within the styles of *PaS_4_-SLFL1 PhS_3_S_3L_*, *PaS_4_-SLFL2 PhS_3_S_3L_*, the parental line *PhS_3_S_3L_*, *PaSFB1 PhS_3_S_3L_*, or *PaSFB4 PhS_3_S_3L_*. Pollen tubes are shown as dotted white lines. Scale bars, 200 μm. E and F, Transgene and *S*-haplotype determination by PCR analysis of self-progeny from transgenic plants of *PaS_4_-SLFL1 PhS_3_S_3L_*, *PaS_4_-SLFL2 PhS_3_S_3L_*, *PaSFB1 PhS_3_S_3L_*, and *PaSFB4 PhS_3_S_3L_*. Wild-type *PhS_3_S_3L_* was used as negative control for transgenes and as positive control for *PhS_3_-RNase* and *PhS_3L_-RNase*. *PhS_3_S_3_* and *PhS_3L_S_3L_* were used as positive or negative controls for corresponding *PhS-RNase*s. ddH_2_O was used as negative control for template DNA.

The *S*-locus of *P.* *avium* (a member of the Rosaceae, representing superrosids) contains two types of *SLF* genes (*SLF* and *SFB* types) (Ushijima et al., 2003). Both types fall within the *SLF* family as described here (Figure  2). To test whether *SLF*s of *Prunus* can detoxify *S-RNase*s in the Solanaceae, we introduced *PaSLFL*s (*SLF* type) and *PaSFB*s (*SFB* type) into the *PhS_3_S_3L_* background (Figure  4, A and B; Supplemental Data Set S3). *PaS_4_-SLFL1* and *PaS_4_-SLFL2* caused breakdown of SI of *P.* *hybrida* and progeny testing indicated that they can inhibit both PhS_3L_-RNase and PhS_3_-RNase (Figure  4, C and E;Supplemental Figure S5; Supplemental Table S3), *PaSFB1* and *PaSFB4* also inhibited both S-RNases (Figure  4, D and F; Supplemental Figure S5; Supplemental Table S3).

**Figure 5:**
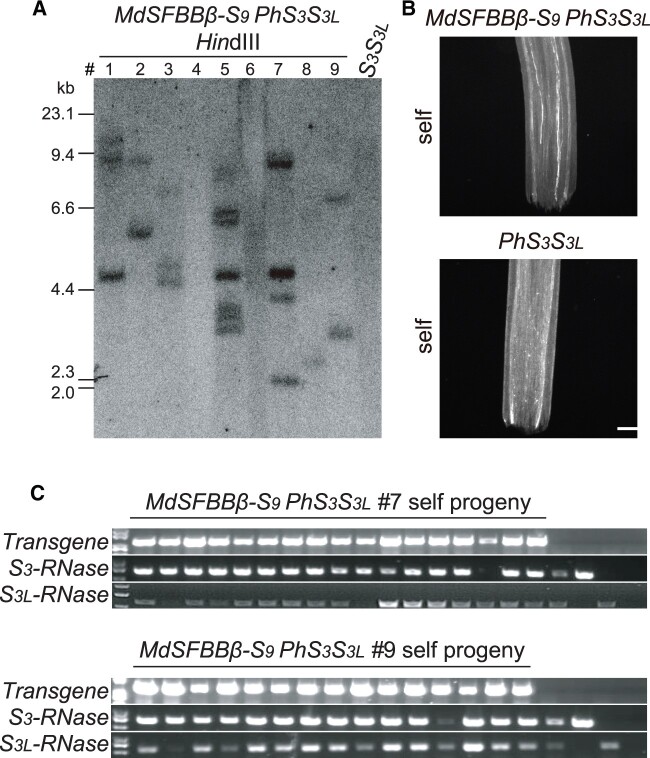
The *MdSFBBβ-S_9_* locus of *M. domestica* functions as the pollen *S* factor. A, Southern blot analysis of T_0_ transgenic lines containing *MdSFBBβ-S_9_*. B, Aniline blue staining of self-pollen tubes from *MdSFBBβ-S_9_ PhS_3_S_3L_* and *PhS_3_S_3L_*. Scale bars, 200 μm. C, Transgene and *S*-haplotype determination by PCR analysis of self-progeny from transgenic plants of *MdSFBBβ-S_9_ PhS_3_S_3L_*. Positive and negative controls are identical to those in Figure  4E.

As a further test of detoxification range, we introduced an *SLF* from another member of the Rosaceae, apple (*Malus domestica*), into *P.* *hybrida.* Its *S*-locus only contains *SLF*-type *SLF*s named *S-locus F-box brother*s (*MdSFBBβ-S_9_*) (Supplemental Data Set S3) and was able to detoxify both PhS_3L_-RNase and PhS_3_-RNase (Figure  5;Supplemental Table S3). Thus, both *SLF*s and *SFB*s of Rosaceae species can detoxify S-RNases in the superasterids.

### *SLF*s and *S-like SLF*s from the Ranunculaceae can detoxify *S-RNase*s in the Solanaceae

**Figure 6:**
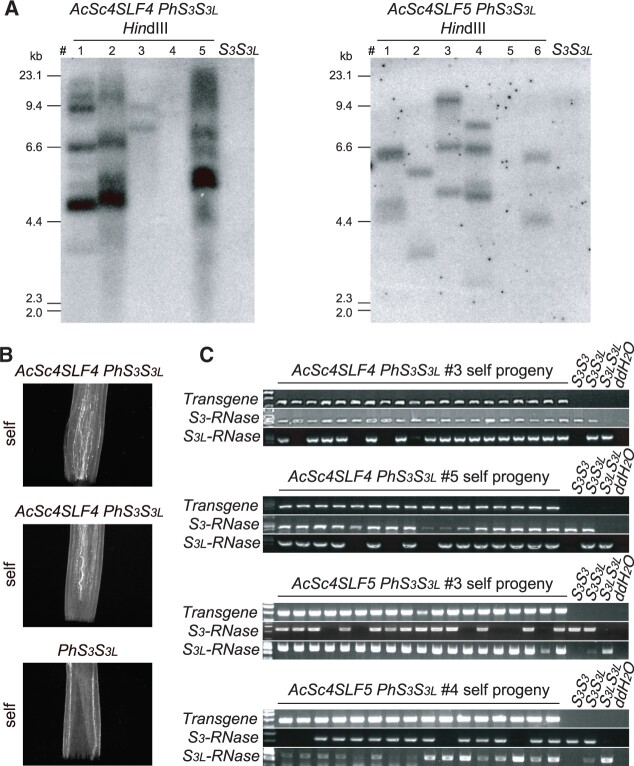
*S-like SLF*s of *A. coerulea* function as the pollen *S* factors. A, Southern blot analysis of T_0_ transgenic lines containing *AcSc4SLF4* and *AcSc4SLF5*. B, Aniline blue staining of self-pollen tubes from *AcSc4SLF4 PhS_3_S_3L_*, *AcSc4SLF5 PhS_3_S_3L_*, and *PhS_3_S_3L_*. Scale bars, 200 μm. C, Transgene and *S*-haplotype determination by PCR analysis of self-progeny from transgenic plants of *AcSc4SLF4 PhS_3_S_3L_* and *AcSc4SLF5 PhS_3_S_3L_*. Positive and negative controls are identical to those in Figure  4E.

To further explore the detoxification range, we annotated homologs of *S-RNase* and *SLF* in Colorado blue columbine (*Aquilegia coerulea*) in the Ranunculaceae (Supplemental Figure S6). *Aquilegia* *coerulea* is cryptically self-incompatible because self-pollen produce fewer seeds than outcross pollen, likely due to inbreeding depression rather than SI (Montalvo, 1992). We detected four type-1 *S*-loci in this species. We introduced two *A. coerulea SLF*s (*AcSc4SLF4* and *AcSc4SLF5*) into the *PhS_3_S_3L_* background (Figure  6A;Supplemental Data Set S3). Both *SLF*s caused breakdown of SI (Figure  6B) and progeny testing indicated that AcSC4-SLF4 can inhibit PhS_3_-RNase, while AcSC4-SLF5 inhibited both PhS_3_-RNase and PhS_3L_-RNase (Figure  6C;Supplemental Table S4). The overall detoxification probability for all *SLFs* tested across taxa was 0.85, which was significantly higher than the probability of 0.186 within *Petunia* (Kubo et al., 2015) (assuming that the expected probability is 5/26 = 0.19, the observed probability is 22/26 = 0.85, χ^2^ test (0.05) = 7.38, *P* = 0.0066).

**Figure 7:**
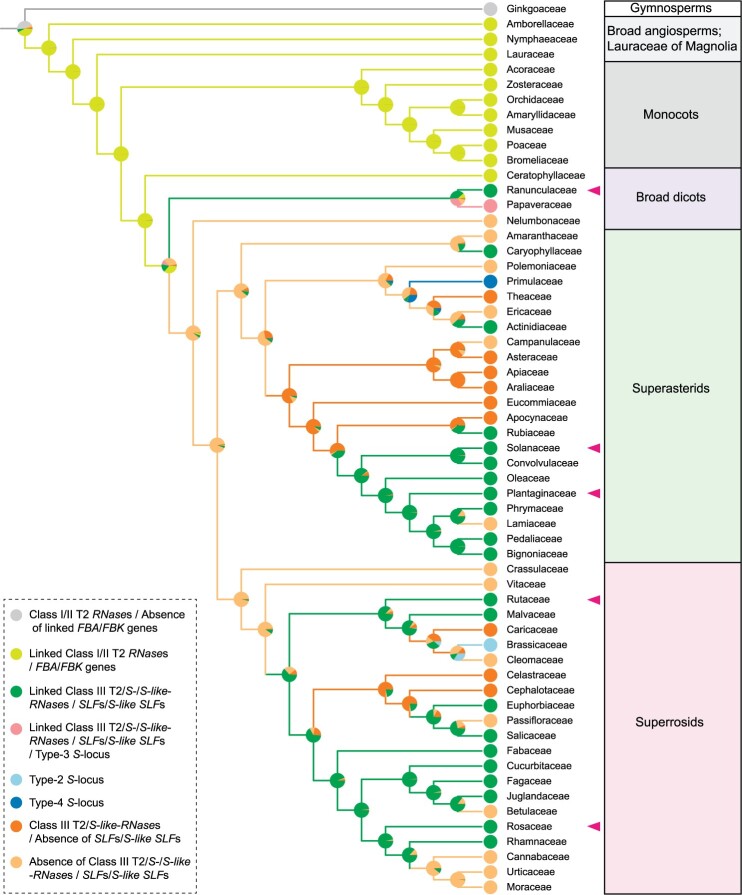
Evolution of the linked Class I/II/III T2 *RNase*s and *FBA*/*FBK* genes inferred by PastML based on a family-level phylogenetic tree of the seed plants. Pie charts indicate probabilities of alternative ancestral states at nodes following analyses of eight traits shown as color-filled circles: possessing Class I/II T2 *RNase*s with no linked *FBA*/*FBK* genes (light gray); possessing loci containing the linked Class I/II T2 *RNase*s and *FBA*/*FBK* genes (light yellow-green); linked Class III T2/*S*-/*S*-*like*-*RNase*s and *SLF*s/*S*-*like SLF*s (green); linked Class III T2/*S*-/*S*-*like*-*RNase*s and *SLF*s/*S*-*like SLF*s/type-3 *S*-locus (pink); type-2 *S*-locus (light blue); type-4 *S*-locus (blue); *S*-*like*-*RNase*s with no linked *SLF*s/*S*-*like SLF*s (orange); and absence of both *S*-/*S*-*like*-*RNase*s and *SLF*s/*S*-*like SLF*s (gold). Colors of the branches are consistent with those of the traits with the highest probabilities. Different evolutionary lineages are illustrated by different color ranges: white for gymnosperms (Ginkgoaceae), light gray for broad angiosperm families and Lauraceae of Magnolia, gray for monocots, purple for broad dicots, green for superasterids, and pink for superrosids. The magenta triangles indicate the five families with genetically demonstrated type-1 *S*-locus.

A ribonuclease T2 family member (Aco001100) tightly linked to several F-box family member genes was identified in pineapple (*A.* *comosus*), indicating that it may have the potential to produce type-1 SI (Chen et al., 2019). Phylogenetic analysis indicated that this T2 RNase groups with Class I rather than Class III T2 RNases (Supplemental Figure S1). In addition to this locus, we identified another region (0.5 Mb) containing tightly linked Class I T2 *RNase* and two *FBK* genes on LG15. We also found three loci containing 1–3 Class I T2 *RNase* and 1–6 *FBK*/*FBA* genes on chromosomes 7–9 in the monocot species rice (Supplemental Figure S7). These genes were expressed constitutively in leaves, anthers (or androecium) and pistils (or gynoecium) (Supplemental Figure S7), indicating a likely expression state of the ancestral type-1 *S*-locus. Pistil-specific (Class III T2/*S*-*RNase*) and pollen-specific (*SLF*s) expression may have evolved later to control eudicot type-1 SI. In the absence of genetic data, it is unclear whether the linked Class I T2 *RNase* and *FBK* genes found in monocots can confer type-1 SI. Ancestral state analysis showed that the common ancestor of angiosperms possessed linked Class I/II T2 *RNase*s and *FBA*/*FBK* genes. Whether the eudicot MRCA had linked Class I/II T2 *RNase*s/*FBA*/*FBK* genes or whether a subfamily evolved toward Class III T2 *RNase*s/*FBA*/*FBK* genes could not be determined with certainty (Figure  7;Supplemental Figure S8).

### Loss and regain of SI

**Figure 8:**
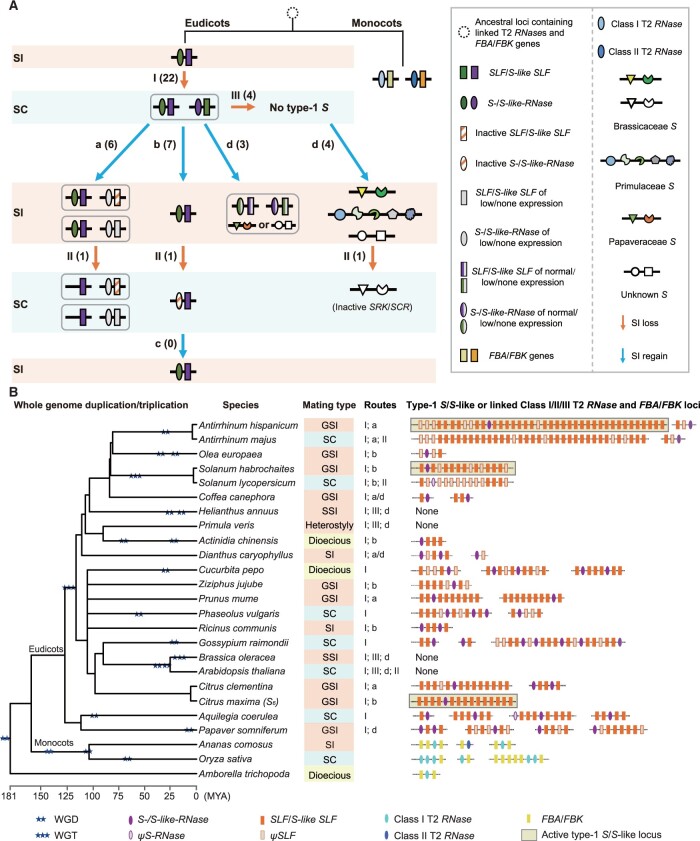
Routes of loss and regain of SI and their associations with type-1 *S-*/*S-*like loci in angiosperm species. A, Seven major routes contributing to the losses of SI leading to SC (I, II, and III) (orange arrow lines) and its regains (a–d) (blue arrow lines) in eudicots. The numbers in parentheses indicate the number of species that has taken each route among 22 eudicot species analyzed in this study. Color-filled rectangles and ovals represent *SLF*/*S*-*like SLF* and *S*-/*S*-*like*-*RNase* genes contributing to type-1 SI, respectively: green and violet for functional genes; light gray for low or no expression; orange stripe-filled for inactive genes. The same color (green or purple) relates to the corresponding *S*-/*S*-*like*-*RNase* and *SLF*/*S*-*like SLF* genes; for example, a green SLF is able to detoxify a green S-RNase. Gray rectangles with curved corners indicate that the *S*/*S-*like-loci are from the same genome. The *S-*loci (*S*) of type-2, -3, and -4 SI are denoted as Brassicaceae *S* (type-2) containing *SRK* and *SCR*, Papaveraceae *S* (type-3) *PrsS* and *PrpS* and Primulaceae *S* (type-4) *CYP*, *GLO*, *CCM*, *PUM*, and *KFB*, respectively. B, Genomic variations of type-1 *S-*/*S*-like loci and the loci of linked Class I/II T2 *RNase*s and *FBA*/*FBK* genes in a total of 25 angiosperm species. Left: phylogenetic tree of eudicot species. Middle: orange, green, and yellow colored background represent GSI, SSI, late-acting SI or heterostyly, SC, and dioecious, respectively. Right: schematic diagrams of annotated genomic structures of linked Class I/II/III T2 *RNase*s and *FBA*/*FBK* loci. Two and three stars depict WGD and WGT, respectively. Ovals filled with different colors indicate *S-*/*S-like-RNase*s (violet), *ψS-RNase*s (light gray), Class I (cyan) or II (blue) T2 *RNase*s and rectangles *SLF*s/*S-like SLF*s (orange), *ψSLF*s (light gray), or *FBA*/*FBK* (yellow) and “None” indicates no type-1 *S*-/*S*-like-locus detected in these species. The numbers and letters labeled in the panel of “Routes” correspond to those indicated in (A).

The early origin of type-1 *S*-locus/SI in eudicots and its ancestral states during the divergence of Nelumbonaceae, superasterids, and superrosids suggest that it has been repeatedly lost during evolution to give SC lineages (Figure  7;Supplemental Figure S8; Fujii et al., 2016). Loss of SI may arise through several routes: (1) Duplication of the *S-*locus to allow two haplotypes within the same genome, causing competitive interaction in the pollen; (2) Deletion or inactivation of components of the *S*-locus (e.g. *S-RNase*); or (3) Deletion of the entire *S-*locus (Figure  8A). Depending on the loss mechanism, SI may then have been regained in some SC lineages through: (1) inactivation or reduced expression of duplicate *S*-loci or *S* genes; (2) deletion of duplicates; (3) reactivation of the *S*-*RNase*; or (4) evolution of a new SI system (Figure  8A). To explore these possible scenarios, we annotated the complete type-1 *S*/*S*-like loci for 22 species representing 18 eudicot families on the basis of genome assembly quality (i.e. containing at least 1-Mb scaffolds, the smallest linkage region for type-1 *S*-/*S*-like loci) (Figure  8B;Supplemental Data Set S6). In addition, we classified type-1 *S-*loci into two types: active *S-*loci, which have putative functional *S-RNase* linked to functional *SLFs*, and inactive *S-*loci in which either the *S-RNase* or *SLF* appeared to be nonfunctional.

Based on the presence of linked T2-type *RNase*s and *FBA*/*FBK* genes in both monocots and eudicots, their common ancestor likely had type-1 SI (Chen et al., 2019), and preceded the diversification of Classes I/II and III T2 *RNase*s. Type-1 SI may have been subsequently lost in many monocots through Routes 1–3. In eudicot lineages, WGDs or triplications (WGT) would have led to duplications of the *S*-locus, causing loss of SI via Route 1 (Figure  8A), raising the question of how SI was maintained in those species that currently exhibit type-1 SI. *Antirrhinum* *hispanicum* contained a duplicate *S-*like locus that comprises a *ψSLF*, an *SLF* expressed to low levels in stamens and an *S-like-RNase* expressed to low levels in styles (Figures  3 and 8, B), indicating that SI was regained via inactivation or reduced expression of duplicate copies (Route a) (Figure  8A). Chinese plum (*Prunus mume*) and clementine (*Citrus × clementina*) also had duplicate loci, one of which may have been degenerated in a similar manner. *Solanum habrochaites*, *C.* *maxima*, olive (*Olea europaea*), red date (*Ziziphus jujube*), and castor bean (*Ricinus communis*) had only one copy of the type-1 *S-*locus, indicating regain of SI via deletion of duplicates (Route b) (Figure  8, A and B).

Two SC species (*A. majus* and *S. lycopersicum*) showed either deletion (*A. majus*) or inactivation (*S. lycopersicum*; Tomato Genome Consortium, 2012) of the *S-RNase* (Route 2). Both species are horticultural varieties that have been selected for SC. Therefore, Route 2 for type-1 SI appears to have been favored in domestication but not in natural populations. Three other SC species, diploid cotton (*Gossypium raimondii*), common bean (*Phaseolus vulgaris*), and *A.* *coerulea* (Montalvo, 1992) had duplicate type-1 *S-*loci, indicating that SI was lost via Route 2 and not regained (Figure  8, A and B).

### Origins of other types of SI in eudicots

We found no evidence of type-1 *S*-like*-*loci in species possessing type-2 (wild cabbage [*Brassica oleracea*]) or type-4 (cowslip [*Primula veris*]) SI (Figure  8B;Supplemental Data Set S6), suggesting ancestral loss of SI through deletion of the type-1 *S-*locus (Route 3) and regain of SI through Route d (Figure  8A).

To examine the origin of type-2 SI, we annotated the pistil (*SRK*s) and pollen (*SCR*s) components in a total of 17 species from 15 genera of the Brassicaceae as well as in spider flower (*Cleome hassleriana*) of the Cleomaceae (Supplemental Figures S9 and S10; Supplemental Data Sets S7 and S8). We only detected *S*-loci, as defined by linkage between the pistil and pollen *S* genes, in the Brassicaceae (Supplemental Figure S9). Nevertheless, we detected *SCR-like* genes (expected threshold cut-off of 100) in upland cotton (*Gosssypium hirsutum*) and cacao (*Theobroma cacao*) of the Malvaceae and *S*. *pennellii* of the Solanaceae, but their linkage to *SRK*-*like* genes was absent (Supplemental Figures S9 and S10). Together with the ancestral state inference analyses of type-2 SI, these results indicated that its evolution occurred in the ancestor of the Brassicaceae (Figure  7). Arabidopsis had no detectable type-1 *S*-locus and its SC likely arose through inactivation of either *SRK* or *SCR* of the *S-*locus via Route 2, as reported previously (Kusaba et al., 2001; Tang et al., 2007; Tsuchimatsu et al., 2010; Figure  8).

To study the origin of type-4 SI, we analyzed the phylogenetic distribution of the *S*-locus supergene described in primrose (*Primula vulgaris*). The clustering of genes in the *S-*locus supergene was found in *P.* *vulgaris* of the Primulaceae but not in species from twelve other families representing monocots and eudicots, including neighboring families (Theaceae, Ericaceae, and Actinidiaceae). These findings suggested that the type-4 *S*-locus supergene evolved only in the Primulaceae (Figure  7;Supplemental Figure S11).

Opium poppy (*Papaver somniferum*), which exhibits type-3 SI, contained four type-1 *S*-like*-*loci, indicating ancestral loss of type-1 SI through duplication (Route 1) and subsequent regain of SI via Route d (Figure  8). To study the origin of type-3 SI, we analyzed the phylogenetic distribution *P.* *rhoeas PrsSs* and *PrpSs* (Supplemental Data Sets S9 and S10)*.* Homologs of *PrsS* were widely detected in eudicots, but we only observed linkage with *PrpS* in the three *Papaveroideae* species examined (*P. rhoeas*, *P. somniferum*, and plume poppy [*Macleaya cordata*]), indicating that type-3 SI evolved in an ancestor of the *Papaveroideae* (Figure  7;Supplemental Figures S12 and S13).

Robusta coffee (*Coffea canephora*), which exhibits GSI, possessed two copies of type-1 *S*-loci, suggesting its regain of SI via either Route a or d (Figure  8). Similarly, common sunflower (*Helianthus annuus*), which exhibits SSI, had no type-1 *S*-like locus, indicating regain of SI via Route d (Figure  8).

## Discussion

Evolution of type-1 SI must satisfy two constraints: (1) SLFs must not detoxify S-RNases from the same haplotype to maintain SI and (2) SLFs must be able to detoxify S-RNases from other haplotypes, allowing cross-compatibility. If the ancestral *S*-locus comprised a single *SLF* and *S-RNase* (Sakai and Haluka, 2014; Sakai, 2016), the first constraint might be readily satisfied by selection for an *S-RNase* that lies outside the detoxification range of the linked *SLF*. Satisfying the second constraint, however, depends on detoxification probability. If this probability is 0.5, as seen with *SLF*s from *Petunia*, pollen would be accepted by only 25% of nonself styles (Kubo et al., 2015). It is difficult to see how the SI system would have evolved with such a strong reproductive cost.

We found that SLFs from diverse taxa can detoxify *Petunia* S-RNases in 22 out of 26 cases, corresponding to a detoxification probability of 0.85. With such a probability, pollen would be accepted by 72% of styles for a single *SLF* linked to an *S-RNase* (Kubo et al., 2015), a much lower reproductive cost than for the probability of 0.5. Thus, we propose that the ancestral system involved SLFs with a high detoxification probability, as observed with our interspecific tests.

Once such a system was established, duplications of the *SLF* would further increase cross-compatibility. For example, pollen with two *SLF*s, each with 0.85 detoxification probability, would be accepted by 96% of styles compared to 72% with only one *SLF* (Kubo et al., 2015). Thus, selection would drive an increase in number of linked *SLF*s. However, there would also be selective pressure to narrow the target range to prevent detoxification within each haplotype (maintaining the first constraint of SI), which may act within each species to reduce target range over evolutionary time, resulting in the currently observed intraspecific detoxification probability of 0.5.

The linkage of *FBA*/*FBK* with T2 *RNase*s likely arose in the common ancestor of monocots and eudicots (Chen et al., 2019). We found that *SLF*s/*S-like SLF*s linked to Class III/*S-RNase*s existed exclusively in eudicots. Thus, if the common ancestor of eudicots and monocots had type-1 SI, this would have preceded the divergence between Classes I/II and III T2 *RNase*s.

Following the origin of type-1 SI, a pattern of losses and gains of SI may be inferred in eudicot lineages (Figure  8). Common routes to loss of SI are inactivation, deletion, or duplication of *S-*loci. Inactivation of the *S-RNase* alone is only observed in domesticated species, suggesting that there may be cost to this route in natural populations, perhaps because the *S-RNase* has a function outside SI. A *Prunus*-specific duplication of *F*-*box* genes has been proposed to generate a functional divergence between *SFB* and *SLFL*/*SFBB* leading to a distinct self-recognition SI system, in which SFB blocks self-S-RNase degradation (Morimoto et al., 2015; Akagi et al., 2016). In support of this self-recognition hypothesis, defective *SFB*s were found to be associated with SC in some *Prunus* species (Hauck et al., 2002; Ushijima et al., 2003, 2004; Tao and Iezzoni 2010). However, our finding that introducing *PaSFB* causes SC (i.e. promotes nonself-S-RNase ubiquitination and degradation) does not support the self-recognition hypothesis. A further observation supporting nonself-recognition rather than self-recognition is competitive interaction between two functional *S*-haplotypes of tetraploid Chinese sour cherry (*Prunus pseudocerasus* Lindl. CV. Nanjing Chuisi) leading to SC (Huang et al., 2008). More recently, the SC of *S4'* pollen containing defective *SFB* was shown to be mediated by *S_4_-SLFL2* (Li et al., 2020). Thus, the validity of the self-recognition hypothesis for *Prunus* remains to be demonstrated.

Type-1 SI has been maintained in many species in the face of WGD through deletion or inactivation of duplicate copies of the *S-*locus. In two cases where type-1 *S-*loci were deleted, SI was regained by the evolution of new SI systems (types -2 and -4). In the case of type-3 SI, four type-1 *S*-like-loci were found in the genome, indicating loss of type-1 SI via duplication. For all SI types, we found that ancestral pistil (e.g. *S-RNase*, *SRK*, *PrsS*, and *CYP*) and pollen (e.g. *SLF*, *SCR*, *PrpS*, and *GLO*) factors, predate the formation of linked SI components. SI may therefore have evolved by duplications and rearrangements bringing the male and female components in tight linkage followed by neofunctionalization or subfunctionalization (Morimoto et al., 2015; Akagi et al., 2016).

Our results reveal a highly dynamic scenario for the evolution of SI. Initial establishment of an ancestral type-1 SI in angiosperms likely involved a single female (T2 *RNase*) and male (*FBK*/*FBA*) component coming into tight linkage. The ancestral single SLF had a high detoxification probability to allow type-1 SI to become established with sufficient cross-compatibility. Selection for further increase in cross-compatibility led to duplication and divergence of the *SLF* within the *S*-locus (Zhou et al., 2003; Kubo et al., 2015; Bod'ová et al., 2018; Li et al., 2019), while selection against detoxification of self-S-RNase led to reduced detoxification probabilities within species. This SI system was then maintained in many lineages despite WGD through deletion of duplicate *S-*loci. In other lineages SI was lost either through deletion or duplication of the *S-*locus. SI was then regained in some cases through novel male and female components coming together to create type-2–4 systems.

## Materials and methods

### Plant materials

Self-incompatible lines of *A.* *hispanicum* and *P.* *hybrida* were derived and maintained as previously described (Xue et al., 1996; Liu et al., 2014). *Solanum habrochaites S_1_S_5_* (LA1777) was obtained from Tomato Genetic Resource Center (University of California, Davis) and *M.* *domestica* and *P.* *avium* from Dr Tianzhong Li’s lab at China Agricultural University (CAU). The partial self-compatible line of *A.* *coerulea* was from Hongzhi Kong’s lab at the Institute of Botany, the Chinese Academy of Sciences (CAS). All materials were planted in a greenhouse at the Institute of Genetics and Developmental Biology, CAS, except *M. domestica* and *P.* *avium*, which were planted in a CAU nursery.

### Molecular techniques

Genomic DNA and total RNA were extracted as previously described (Lai et al., 2002; Liu et al., 2014). First-strand cDNAs were synthesized using SuperScript reverse transcriptase (Invitrogen, Carlsbad, MA, USA). The isolation of the coding sequences for *AhSLF*s, *PaSLFL*s, and *PaSFB*s were described previously (Zhou et al., 2003; Ikeda et al., 2004; Vaughan et al., 2006). The coding sequences for *ShSLF*s, *AcSLF*s, and *MdSFBB* were isolated from anther cDNAs of their source species. Restriction sites for BglII and SmaI were respectively introduced at the 5′- or 3′-end of *AhSLF*s and for XbaI and SacI at the 5′- or 3′-end of other *SLF*s. All *SLF*s coding sequences were ligated into modified pBI101 vectors as described previously (Qiao et al., 2004b; Liu et al., 2014) in which the tomato pollen-specific *LAT52* promoter was used to express *AhSLF*s; the *PhS_3_A-SLF1* promoter was used for other *SLF*s. The accession numbers of *SLF*s and the primers used in this study are listed in Supplemental Data Sets S11 and S12, respectively.

### Transformation of *P. hybrida*

The vectors containing *SLF*s were individually introduced into Agrobacterium (*Agrobacterium tumefaciens*) strain LBA4404 (Invitrogen, Carlsbad, CA, USA; http://www.thermofisher.com/) by electroporation and transformed into leaf disks of *P.* *hybrida PhS_3_S_3L_* as previously described (Lee et al., 1994; Qiao et al., 2004b). Southern blotting analysis was used to detect the transgenes. Briefly, genomic DNA (10 μg) was first digested with HindIII at 37°C for 4 h, then digested overnight after adding fresh HindIII. The DNA fragments were separated by electrophoresis overnight at 1 V/cm on a 0.8% (w/v) agarose gel and the separated fragments were transferred onto Hybond N+ nylon membranes (Amersham, Buckinghamshire, UK, http://www.gelifesciences.com/). *Neomycin Phosphotransferase 2* (*NPTII*) probes were labeled with ^32^P using the Prime-a-Gene labeling system (Promega, Madison, WI, USA; https://www.promega.com/). The pre-hybridization, hybridization and membrane washing steps were based on the operation manual (Russell and Sambrook, 2001). Radioactive signals were detected with a phosphor screen and a multifunctional laser scanning imager (GE Typhoon FLA9500).

### Pollination analysis of transgenic plants

Self-pollination was performed using open flowers covered with paper bags before and after pollination to prevent cross pollination. Seedlings of one mature capsule from self-pollination for each transgenic line were used for genotyping. To examine the inheritance of *SLF* transgenes and the *S*-haplotypes of the progeny, PCRs with genomic DNA were performed using gene-specific primers listed in Supplemental Data Set S12. After amplification for 30 cycles, the products were separated by gel electrophoresis and detected by ethidium bromide staining.

### Aniline blue staining of pollen tubes

About 48 h after self-pollination of transgenic plants and the wild-type *PhS_3_S_3L_*, the pollinated styles were fixed in ethanol: glacial acetic acid (3:1, v/v) solution for at least 8 h. Aniline blue staining of pollen tubes was then performed as described previously (Liu et al., 2014; Li et al., 2017) and the stained pollen tubes were observed under ultraviolet light by fluorescence microscopy.

### Construction of family-/species-level phylogenetic trees and estimation of divergence times

Family-/species-level phylogenetic trees were constructed by phylomatic (version 3) (http://phylodiversity.net/phylomatic/) with the divergence times in those trees derived from TimeTree (http://www.timetree.org/) and WGD/WGTs from previous studies (Jiao et al., 2014; McKain et al., 2016; Leebens-Mack et al., 2019). Evolview (version 2) (http://www.evolgenius.info/evolview; He et al., 2016) and MEGAX (Sudhir et al., 2018) were used for visualization, annotation and management of the trees.

### Annotations of the type-1 S-loci, type-1 S-like-loci and type-4 S-like-locus supergene

The genomic structures of the type-1 *S*-loci and type-1 *S*-like-loci were annotated based on the phylogenetic analyses of both Class III T2 *RNase*s and their linked *SLF*s/*S*-like *SLF*s, except for the *S*-locus of *A.* *majus* that was described previously (Li et al., 2019). *ψS-RNase*s and *ψSLF*s were annotated with in-frame stop codons. Based on the results of TBLASTN of the type-4 *S*-locus supergene, their homologs were searched within a 1-Mb window in the genomes of species other than Primulaceae with tandem repeats and reverse order allowed.

### Phylogenetic analysis of the *S* and *S*-like genes

We obtained amino acid sequences corresponding to *S*-like genes by performing BLASTP or TBLASTN with BLAST version 2.2.29 against the protein or genome databases of the seed plants listed in Supplemental Data Set S13 using amino sequences of the proteins encoded by known *S* genes as queries. Databases were created (command: makeblastdb.exe -in filename.fasta -parse_seqids -hash_index -dbtype nucl/prot). BLAST was run using default settings with the expected threshold cut-off of 10^−5^ (homologous genes of *PUM*, *GLO*, *CYP*, *KFB* and *CCM*), 10^−6^ (T2 *RNase*s, *FBA*s/*FBK*s, and *PrsS*/*PrsS-like*s), 0.05 (*SCR/SCR-like*s and *PrpS/PrpS-like*s), and 0 (*SRK/SRK-like*s), respectively. In addition, BLAST for FBAs/FBKs was only performed in 33 species containing Class III T2 RNases except for *O.* *sativa*. Protein sequences obtained from BLAST were screened by InterProScan version 5.36 (Jones et al., 2014) (command: sh interproscan.sh -appl PfamA, TIGRFAM, SMART, SuperFamily, PRINTS -dp -f tsv, html –goterms –ipr lookup -t p -i filename.fa) and sequences containing a T2 RNase family or FBA1/FBA3 domain were retained. Then only *FBA*s/*FBK*s located near Class III T2 *RNase*s (upstream or downstream within 3 Mb) were obtained, except for species without such *FBA*s/*FBK*s. The full-length protein sequences of T2 RNases and FBAs/FBKs were aligned using L-INS-i method in MAFFT version 7.407 (Katoh and Standley, 2013) (command: nohup mafft –localpair –maxiterate 1000 –thread 64 $1 > "$NAME".afa &) and other proteins encoded by *S* genes using Muscle in MEGAX. We then manually curated the alignments using AliView (Larsson, 2014) or MEGAX to delete gaps and sequences without conserved motifs. To construct maximum likelihood (ML) trees, best-fitting amino acid substitution models were determined using ModelFinder within IQtree (-m MF -msub nuclear -nt AUTO) (Kalyaanamoorthy et al., 2017): a VT + R5 model for T2 RNase superfamily of the four exemplar families (Plantaginaceae, Solanaceae, Rutaceae, and Rosaceae), JTT + F + R7 for FBAs/FBKs of the four exemplar families, WAG + F + R4 for T2 RNases of *A.* *coerulea* and S-RNases of the four exemplar families, JTT + F + R5 for FBAs/FBKs of *A. coerulea* and SLFs of the four exemplar families, JTT + R4 for SRKs/SRK-likes, VT + R3 for SCRs/SCR-likes, VT + R6 for PrsSs/PrsS-likes, JTT + G4 for PrpSs/PrpS-likes, WAG + G for T2 RNases and VT + F + R10 for FBAs/FBKs. Then ML trees were inferred using IQ-TREE with default settings under the previously determined best-fitting amino acid substitution model. We measured branch support using the Ultrafast Bootstrap [UFBoot] algorithm with 1,000 replicates. MEGAX and iTOL (https://itol.embl.de/) was used for visualization, annotation and management of the trees.

### Transcriptome analysis

Total RNA was extracted from four *A.* *hispanicum* tissues (leaf, pistil, stamen, and petal) using RNAprep Pure Plant Kit (Tiangen, Beijing, China). RNA purity and integrity were assessed using a NanoPhotometer^®^ spectrophotometer (IMPLEN, CA, USA) and RNA Nano 6000 Assay Kit on a Bioanalyzer 2100 system (Agilent Technologies, Santa Clara, CA, USA), respectively. Sequencing libraries were generated using NEBNext UltraTM RNA Library Prep Kit for Illumina (NEB, USA). All libraries were sequenced using Illumina HiSeq 2000 (2 × 100 bp). Raw reads were quality-checked with FastQC96 (version 0.11.8) (http://www.bioinformatics.babraham.ac.uk/projects/fastqc; Andrews, 2010) and the resulting clean reads were aligned to the *A*. *hispanicum* genome (https://ngdc.cncb.ac.cn/gsa/browse, genome warehouse (GWH) accession GWHBFSA00000000, BioProject ID PRJCA006945) using STAR (version 2.7.1a) (Dobin et al., 2013) with parameters “-alignIntronMax 6000 -alignIntronMin 50” and the expression quantification for each gene was performed using RNA-seq by expectation maximization (RSEM) (Li and Dewey, 2011). The genome sequencing and assembly of *A*. *hispanicum* were performed as described by Li et al. (2019). The RNA-seq analyses of rice and pineapple T2 *RNase*s and *FBA*/*FBK* genes were performed based on the expression matrix data in Rice Genome Annotation Project and EBI-ENA under the accession number PRJEB33121 (Chen et al., 2019), respectively.

### Ancestral state reconstruction

To evaluate the evolution of type-1 *S*-locus, five discrete traits were selected and codified as: possessing Class I/II T2 *RNase*s with no linked *FBA*/*FBK* genes; linked Class I/II T2 *RNase*s and *FBA*/*FBK* genes; linked Class III T2/*S*-/*S*-*like*-*RNase*s and *SLF*s/*S*-*like SLF*s; *S*-*like*-*RNase*s with no linked *SLF*s/*S*-*like SLF*s; and absence of both *S*-/*S*-*like*-*RNase*s and *SLF*s/*S*-*like SLF*s. Three additional traits (possession of type-2, type-3, or type-4 *S*-locus) were selected to reconstruct the evolution of these three types of *S*-loci as well as the evolutionary relationship of the four types of *S*-loci. Ancestral state reconstruction was conducted using PastML (https://pastml.pasteur.fr/help; Ishikawa et al., 2019) based on a family-level phylogenetic tree constructed as described above. ML method marginal posterior probabilities approximation under F81 model (Felsenstein, 1981) was used as recommended (Ishikawa et al., 2019) and iTOL (https://itol.embl.de/) was used for visualization and annotation of the results.

### Calculation of probabilities for S-RNase detoxification by SLFs

To estimate the detoxification probability of n SLF types (*P_n_*), we applied the equation “*P_n_* = 1 - (1 - *P_R_*) ^n^” reported in Kubo et al. (2015) assuming that S-RNase is independently recognized by each SLF with the same probability *P_R_*. According to previous studies, a single SLF can recognize about 50% (i.e. *P_R_* = 0.5) (Supplemental Data Set S3) or less (Kubo et al., 2015) S-RNases from the same species and the detoxification probability of pollen with a single SLF is 0.5 (i.e. *P_n_* (*n* = 1) = 0.5) or less (Supplemental Data Set S3; Kubo et al., 2015). As SLFs from different species can detoxify *Petunia* S-RNases in 22/26 cases, the recognition probability is 0.85 (22/26) (i.e. *P_R_* = 0.85) and pollen with a single SLF can detoxify 85% of S-RNases (i.e. *P_n_* (*n* = 1) = 0.85) (Kubo et al., 2015), thus accepted by 72% (i.e. 0.85^2^) of styles (assuming females are heterozygous and thus carrying two S-RNases to be detoxified). Likewise, pollen with two SLFs, each with 0.85 (*P_R_*) recognition probability, would have a detoxification probability of 0.98 (i.e. *P_n_* (*n* = 2) = 0.98) (Kubo et al., 2015) and would be accepted by 96% (i.e. 0.98^2^) of females.

### Accession numbers

All sequence data generated in the context of this manuscript have been deposited in the China National Center for Bioinformation Genome Sequence Archive database (https://ngdc.cncb.ac.cn/gsa/browse; Chen et al., 2021; CNCB-NGDC Members and Partners, 2021): Illumina reads for RNA-seq in the Genome Sequence Archive database (GSA accession CRA005238, BioProject ID PRJCA006940) and the whole-genome sequence data and assemblies of *A.* *hispanicum* in the Genome Warehouse database (GWH accession GWHBFSA00000000, BioProject ID PRJCA006945).

## Supplemental data 

The following materials are available in the online version of this article.

**Supplemental Figure S1.** ML tree of the T2 RNase superfamily of seed plants.

**Supplemental Figure S2.** ML tree of the FBA/FBKs of seed plants.

**Supplemental Figure S3.** *SLF*s of *S. habrochaites* function as pollen *S* factors.

**Supplemental Figure S4.** Both *SLF*s from the *S*-locus of *A.* *hispanicum* and an *SLFL* from an *S*-like-locus of *A*. *majus* function as the pollen *S* factors.

**Supplemental Figure S5.** Southern blot analysis of self-progeny plants of *PaS_4_*-*SLFL1 PhS_3_S_3L_*, *PaS_4_*-*SLFL2 PhS_3_S_3L_*, *PaSFB1 PhS_3_S_3L_*, and *PaSFB4 PhS_3_S_3L_*.

**Supplemental Figure S6.** Phylogenetic analyses of the *S* genes of *A.* *coerulea*.

**Supplemental Figure S7.** Transcript profiles of T2 *RNase*s and their linked *FBA*/*FBK* genes in *O.* *sativa* and *A.* *comosus*.

**Supplemental Figure S8.** Evolution of SI systems and their *S* genes in a family-level phylogenetic tree of the seed plants.

**Supplemental Figure S9.** The evolution of type-2 SI system in angiosperms.

**Supplemental Figure S10.** Phylogenetic analyses of the *S* genes of type-2 SI.

**Supplemental Figure S11.** The evolution of type-4 SI system in angiosperms.

**Supplemental Figure S12.** The evolution of type-3 SI system in angiosperms.

**Supplemental Figure S13.** Phylogenetic analyses of *S* genes of type-3 SI.

**Supplemental Table S1.** Pollination and genotype analyses of transgenic plants of *ShSLF*s *PhS_3_S_3L_*.

**Supplemental Table S2.** Pollination and genotype analyses of transgenic plants of *AhSLF*s *PhS_3_S_3L_* and *AmSLFL PhS_3_S_3L_*.

**Supplemental Table S3.** Pollination and genotype analyses of transgenic plants of *PaSLFL*s *PhS_3_S_3L_*, *PaSFB*s *PhS_3_S_3L_*, and *MdSFBB PhS_3_S_3L_*.

**Supplemental Table S4.** Pollination and genotype analyses of transgenic plants of *AcSLF*s *PhS_3_S_3L_*.

**Supplemental Data Set S1.** Accession numbers of T2 *RNase* genes used in this study.

**Supplemental Data Set S2.** Accession numbers of *FBX*/*FBA*/*FBK* genes used in this study.

**Supplemental Data Set S3.** Summary of species, *SLF*s/*SLFL*s/*SFB*s used for transformation, and *S* haplotypes of compatible transgenic pollen caused by competitive interaction.

**Supplemental Data Set S4.** RNA-seq data of the *S*-locus in four tissues of *A.* *hispanicum* used in this study.

**Supplemental Data Set S5.** RNA-seq data of the *S*-like-locus in four tissues of *A.* *hispanicum* used in this study.

**Supplemental Data Set S6.** Species annotated with linked pistil-pollen gene pairs in this study.

**Supplemental Data Set S7.** Accession numbers of *SRK*/*SRKL*, genes used in this study.

**Supplemental Data Set S8.** Accession numbers of *SCR*/annotated *SCR* genes used in this study.

**Supplemental Data Set S9.** Accession numbers of *PrsS*/annotated *PrsS* genes used in this study.

**Supplemental Data Set S10.** Accession numbers of *PrpS*/annotated *PrpS* genes used in this study.

**Supplemental Data Set S11.** Accession numbers of transgenic *FBX*/*SLF* genes used in this study.

**Supplemental Data Set S12.** Primer list.

**Supplemental Data Set S13.** Genome databases used in this study.

**Supplemental File S1.** Fasta files of the sequence alignments corresponding to the phylogenetic analyses of the type-1–4 *S* genes.

**Supplemental File S2.** Tree text files corresponding to the phylogenetic analyses in Figures  1, 2, 7, and 8 and Supplemental FiguresS1, S2, S6, and S8–S13.

## Supplementary Material

koab266_Supplementary_Data

## Notes

These authors contributed equally to this work (Ho.Z., Y.Z., Hu.Z., Y.S., F.Z., Y.Z., and S.Z.).

Senior author.

Ho.Z. and Y.Z. performed the phylogenetic analyses of all the *S* genes and *S*-*like* genes. Y.Z. performed the functional analyses of *SLF*s of Rosaceae and Ranunculaceae. Hu.Z. and S.Z. performed the phylogenetic analyses of the *S* genes and *S*-*like* genes of type-1 SI. Y.S. performed the functional analyses of *AhSLF*s, *AmSLF*, and *AmFBX*. F.Z., Y.E.Z., Y.J.Z., and Q.G. performed genome analysis of *Antirrhinum hispanicum*. Ho.Z. and S.Z. performed ancestral state reconstruction analyses of SI systems, the genomic structure analyses of the linked Class I/II T2 *RNase*s and *FBA*/*FBK* loci of monocots and the related RNA-seq analyses, and H.K.Z assisted the RNA-seq analyses. Y.E.Z. and S.Z. performed transcriptional analyses of *A*. *hispanicum S*- and *S*-like-locus. Z.Z. and H.C. assisted the phylogenetic analyses of all the *S* genes and *S*-like genes. J.L. assisted the functional analysis of *SLF*s of Rosaceae. Ho.Z., H.H., and Q.L. assisted the functional analyses of *AhSLF*s. H.G. and M.L. performed functional analyses of *ShSLF*s. Q.H., L.C., and Y.E.Z prepared plant materials. Ho.Z., Y.Z., Hu.Z., F.Z., Z.Z., Q.G., H.C., E.C., Y.J.Z., and Y.X. analyzed data. E.C. conceived the idea of a high detoxification probability accounting for the origin of type-1 SI. Y.X. conceived the project and designed experiments. Ho.Z., Y.Z., E.C., and Y.X. wrote the manuscript with the input of all other authors.

The author responsible for distribution of materials integral to the findings presented in this article in accordance with the policy described in the Instructions for Authors (https://academic.oup.com/plcell) are: Yongbiao Xue (ybxue@genetics.ac.cn).
