# Supplementary material for: Origin, loss, and regain of self-incompatibility in angiosperms
Source: Plant Cell. 2021 Nov 4;34(1):579–96. doi: 10.1093/plcell/koab266 (PMC8774079; doi:10.1093/plcell/koab266)
Supplement: koab266_Supplementary_Data [file koab266_supplementary_data.zip › TPC2021RA00601DR1 Supplemental Figures and Tables.pdf]

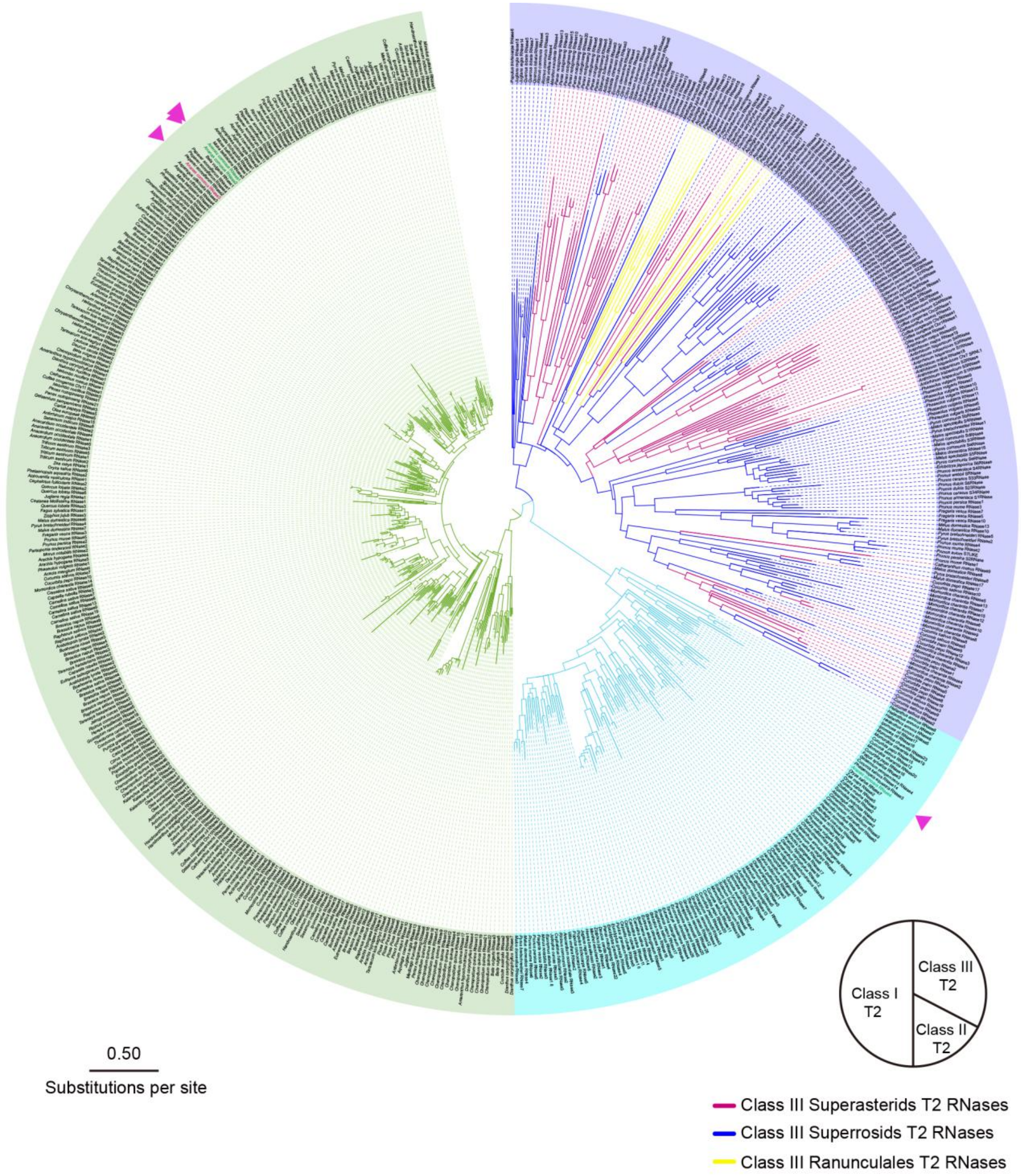

Supplemental Figure S1. Maximum-likelihood tree of the T2 RNase superfamily in seed plants.

Maximum-likelihood phylogenetic tree of T2 RNases from 102 species of *Pinus* (gymnosperms), angiosperms, monocot and eudicot plants. The T2 RNases of the three classes (I, II and III) are labeled by different color ranges, with the Class III T2 RNases from different evolutionary lineages (superasterids, superrosids and Ranunculales) illustrated by different branch colors. The four magenta triangles indicate four annotated T2 RNases in pineapple (*Ananas comosus*). The pineapple T2 RNase Aco001100 is shown in red; the other three RNases are shown in green. Please refer to Supplemental File S2 for the detailed bootstrap values.

(Supports Figures 1 and 7).

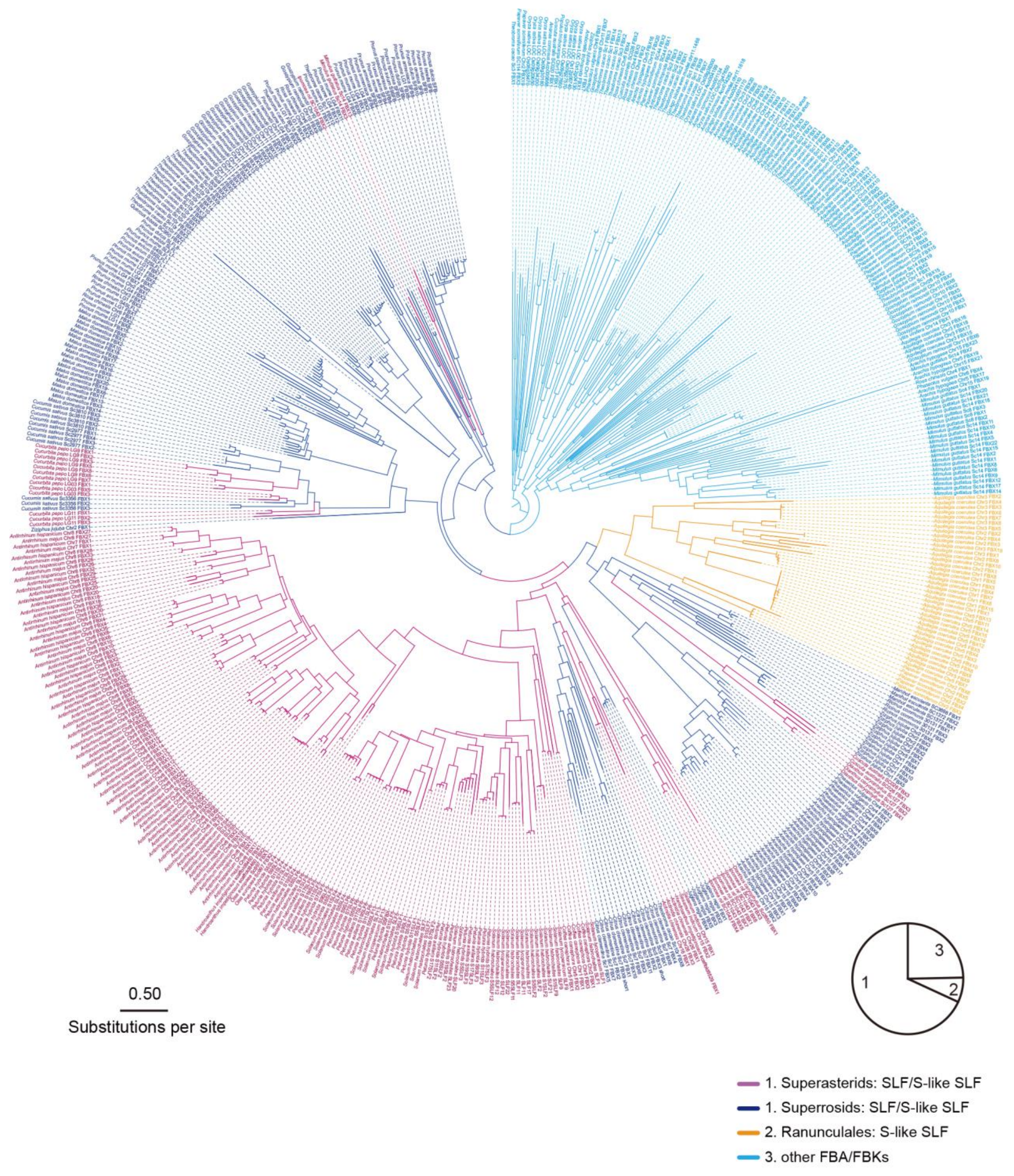

Supplemental Figure S2. Maximum-likelihood tree of the FBA/FBKs of the seed plants.

Maximum-likelihood phylogenetic tree of FBAs/FBKs from 42 species of gymnosperm, angiosperm, monocot and eudicot plants. Functional and *S*-like SLFs from different evolutionary lineages are indicated by different branch colors. Please refer to Supplemental File S2 for the detailed bootstrap values.

(Supports Figures 2 and 7).

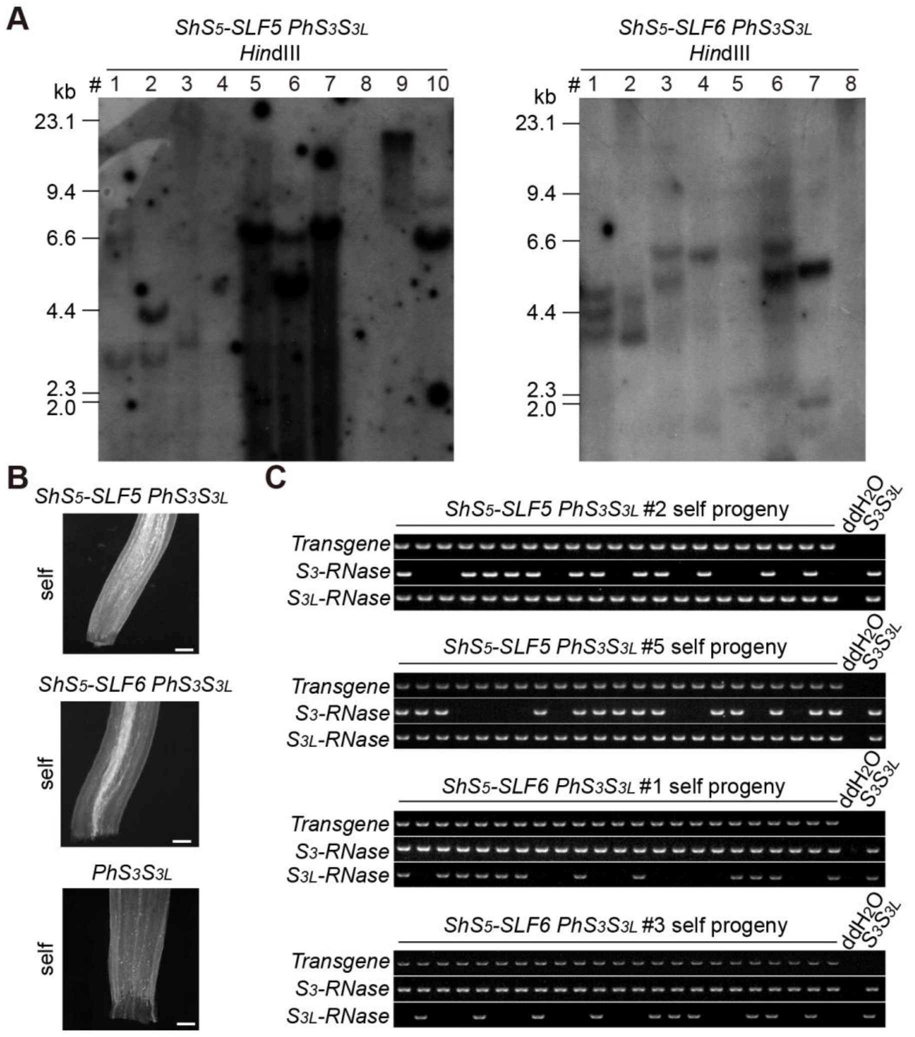

Supplemental Figure S3. *SLF*s of *Solanum habrochaites* function as the pollen *S* factors.

(A) Southern blot analysis of *Petunia* transgenic lines containing *ShS*$_5$*-SLF5* and *ShS*$_5$*-SLF6*.

(B) Aniline blue staining of self pollen tubes from *ShS*$_5$*-SLF5 PhS*$_3$*S*$_{3L}$, *ShS*$_5$*-SLF6 PhS*$_3$*S*$_{3L}$ and *PhS*$_3$*S*$_{3L}$. Scale bars, 200 μm.

(C) Transgene and *S*-haplotype determination by PCR analysis of self-progeny from transgenic plants of *ShS*$_5$*-SLF5 PhS*$_3$*S*$_{3L}$ and *ShS*$_5$*-SLF6 PhS*$_3$*S*$_{3L}$. Wild-type *PhS*$_3$*S*$_{3L}$ was used as negative control for transgenes and as positive control for *PhS*$_3$*-RNase* and *PhS*$_{3L}$*-RNase*. ddH$_2$O was used as negative control for template DNA.

(Supports Figures 4, 5 and 6).

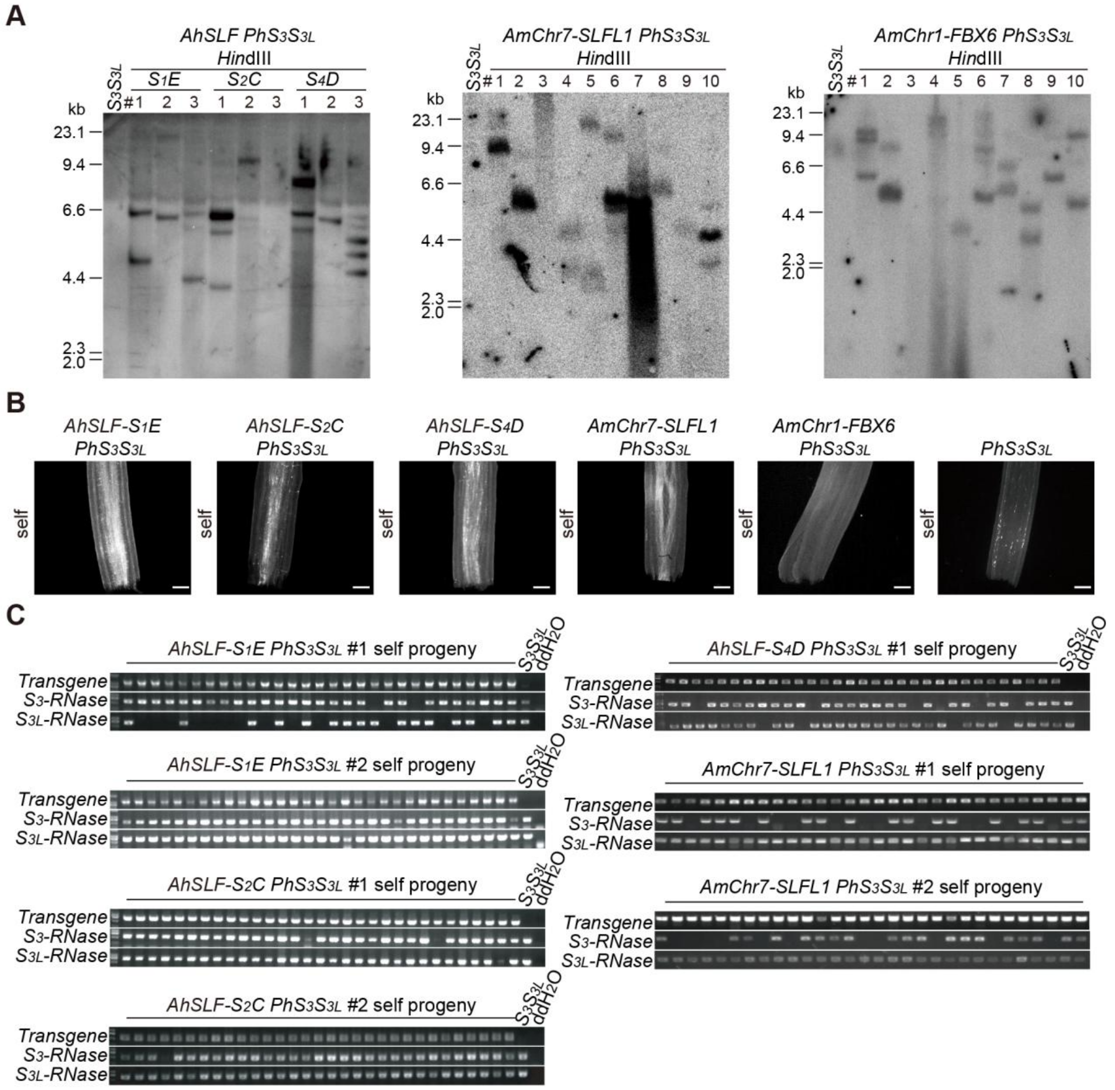

Supplemental Figure S4. Both *SLF*s from the *S*-locus of *Antirrhinum hispanicum* and an *SLFL* from an *S*-like-locus of *A. majus* function as the pollen *S* factors.

(A) Southern blot analysis of T_0 transgenic lines containing *AhSLF-S_1E*, *AhSLF-S_2C*, *AhSLF-S_4D*, *AmChr7-SLFL1* and *AmChr1-FBX6*.

(B) Aniline blue staining of self pollen tubes from *AhSLF-S_1E PhS_3S_{3L}*, *AhSLF-S_2C PhS_3S_{3L}*, *AhSLF-S_4D PhS_3S_{3L}*, *AmChr7-SLFL1 PhS_3S_{3L}*, *AmChr1-FBX6 PhS_3S_{3L}* and *PhS_3S_{3L}*. Scale bars, 200 μm.

(C) Transgene and *S*-haplotype determination by PCR analysis of self-progeny from transgenic plants of *AhSLF-S_1E PhS_3S_{3L}*, *AhSLF-S_2C PhS_3S_{3L}*, *AhSLF-S_4D PhS_3S_{3L}* and *AmChr7-SLFL1 PhS_3S_{3L}*. Positive and negative controls are identical to those in Supplemental Figure S3.

(Supports Figures 4, 5 and 6).

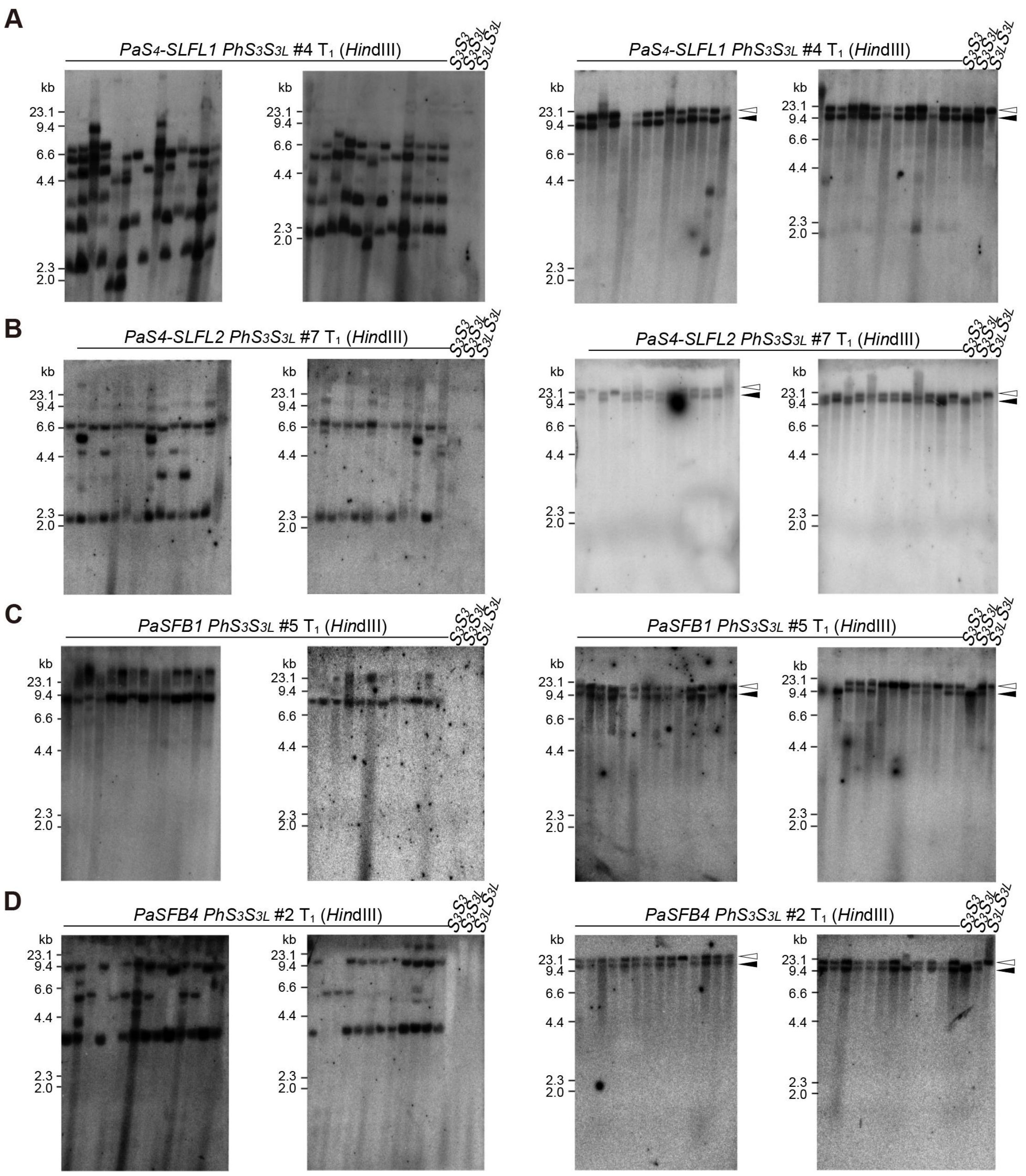

Supplemental Figure S5. Southern blot analysis of self-progeny plants of *PaS₄-SLFL1 PhS₃S₃L*, *PaS₄-SLFL2 PhS₃S₃L*, *PaSFB1 PhS₃S₃L* and *PaSFB4 PhS₃S₃L*.

(A-D) The genomic DNA of transgenic lines and wild type *Ph*S_3S_3, *Ph*S_3S_{3L} and *Ph*$S_{3L}S_{3L}$ was thoroughly digested by *Hin*d III. ^{32}P-labeled *NPTII*, *Ph*S_3*-RNase* and *Ph*S_{3L}*-RNase* were used as probes to detect transgenes (left) and *S*-haplotypes (right). Left: wild-type *Ph*S_3S_3, *Ph*S_3S_{3L} and *Ph*$S_{3L}S_{3L}$ were used as negative controls. Right: the open black and black triangles indicate the DNA fragments from *Ph*S_{3L}*-RNase* and *Ph*S_3*-RNase*, respectively. Wild-type *Ph*S_3S_{3L} was used as positive controls of both *Ph*S_{3L}*-RNase* and *Ph*S_3*-RNase*. Wild type *Ph*S_3S_3 was used as the positive control of *Ph*S_3*-RNase* and the negative control of *Ph*S_{3L}*-RNase* while wild type *Ph*$S_{3L}S_{3L}$ was used as the positive control of *Ph*S_{3L}*-RNase* and the negative control of *Ph*S_3*-RNase*.

(Supports Figure 4).

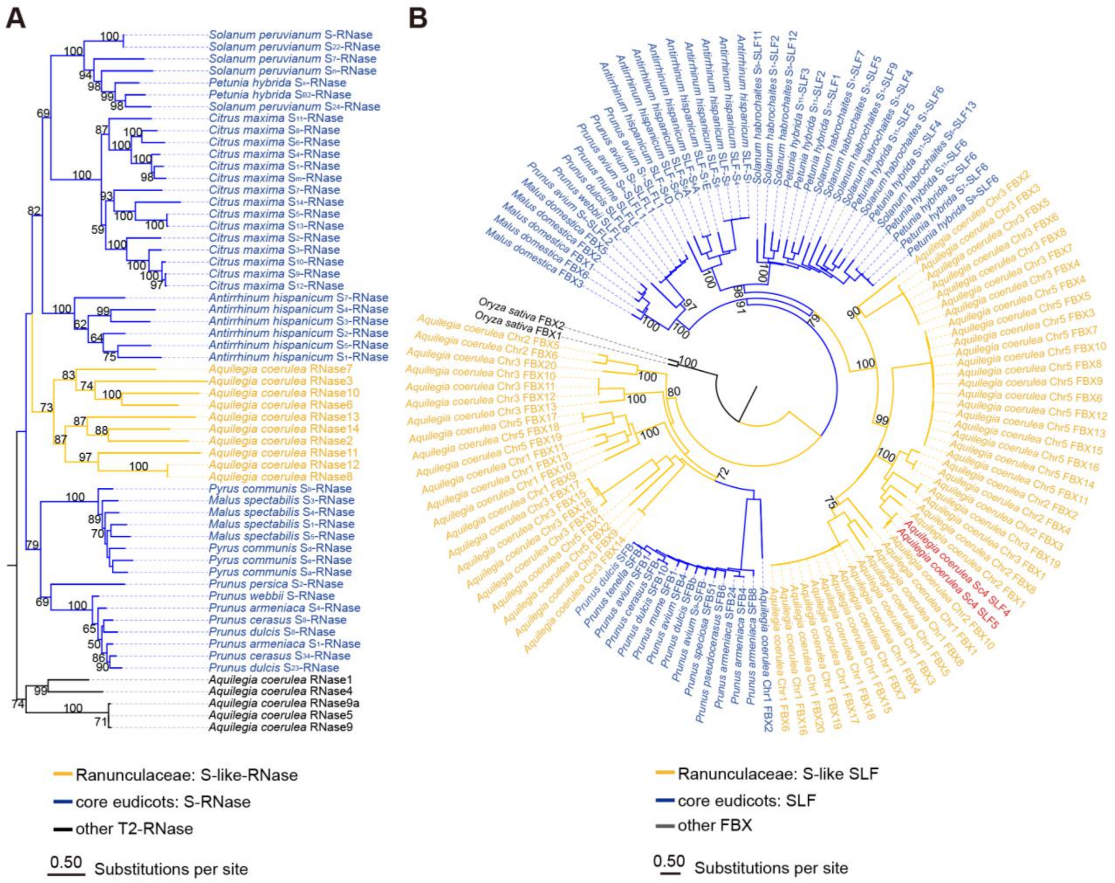

Supplemental Figure S6. Phylogenetic analyses of the *S* genes of *Aquilegia coerulea*.

(A) Maximum-likelihood tree of T2 RNases of *Aquilegia coerulea* and S-RNases of the four exemplar families with some bootstrap confidence values above 50% are shown. The S-/S-like-RNases from different families and other types of T2 RNases are indicated by different branch colors.

(B) Maximum-likelihood tree of FBA/FBKs of *A. coerulea* and SLFs of the four exemplar families with those from *Oryza sativa* as a root and some bootstrap confidence values above 50% shown in the tree. The SLFs/S-like SLFs from different families and other FBA/FBKs are indicated by different branch colors. Red letters indicate genes used for functional analysis.

Please refer to Supplemental File S2 for the detailed bootstrap values.

(Supports Figure 6).

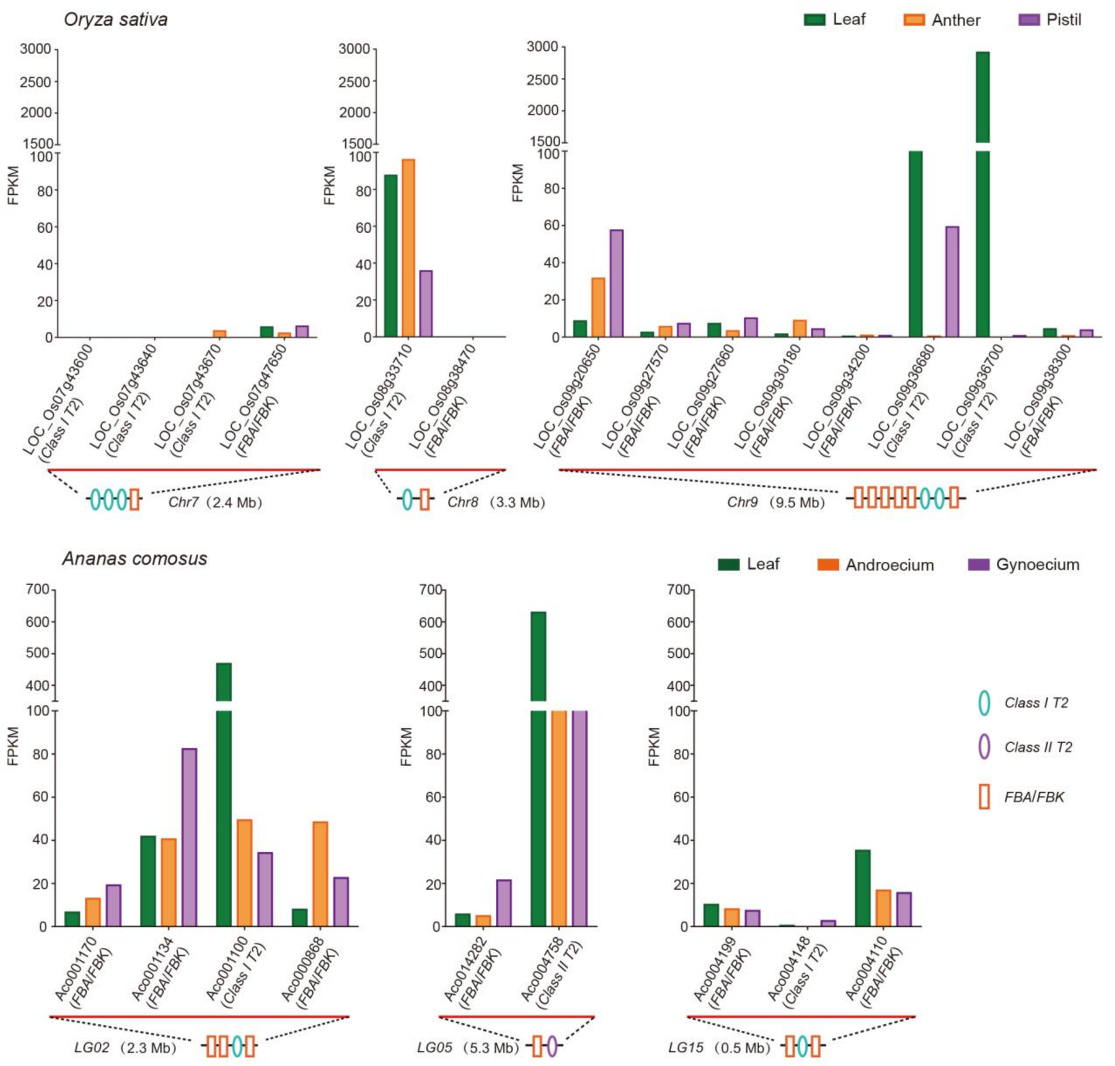

Supplemental Figure S7. Transcript profiles of T2 *RNase*s and their linked *FBA*/*FBK* genes in *Oryza sativa* and *Ananas comosus*

Fragments Per Kilobase of transcript per Million mapped reads (FPKM) of annotated T2 *RNase* and *FBK*/*FBA* expression in leaf, anther and pistil of *O. sativa* (top) or in leaf, androecium and gynoecium of *A. comosus* (bottom). Cyan and violet ovals indicate T2 *RNase* genes encoding Class I and Class II T2 RNases, respectively, and orange rectangles the *FBA*/*FBK* linked with T2 *RNase* genes. Chr: chromosome; LG: linkage group.

(Supports Figure 8).

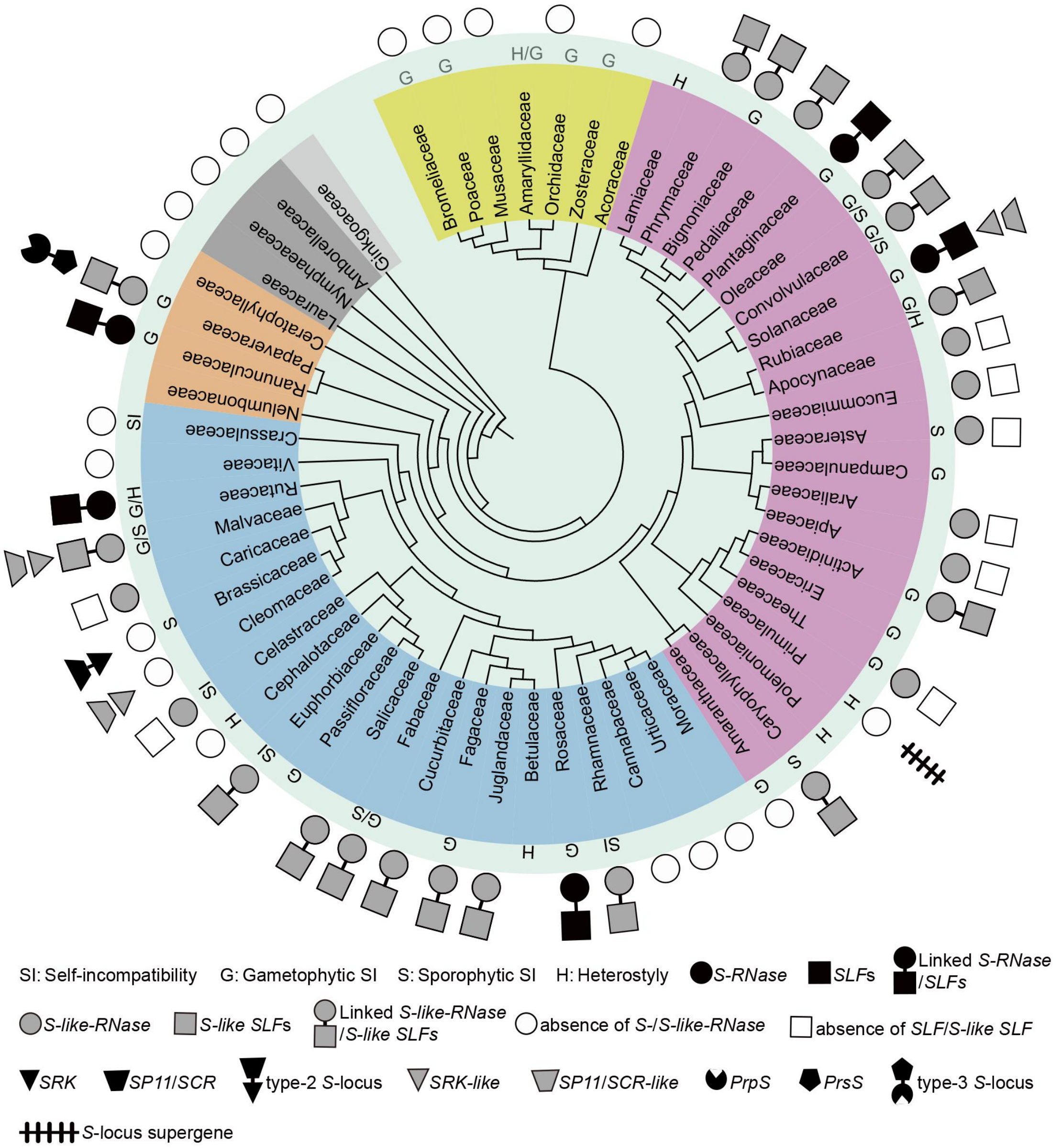

Supplemental Figure S8. Evolution of SI systems and their *S* genes in a family-level phylogenetic tree of the seed plants.

The tree in the center represents a phylogenetic tree of 59 angiosperm families and Pinaceae. The light gray color indicates the gymnosperms (Ginkgoaceae), gray the broad families of the angiosperms and the Lauraceae of Magnolia, yellow monocots, orange basal dicots, blue superrosids and violet superasterids. *S* genes (black), *S-like* genes (gray) or none *S-*/*S-like-RNase*s (hollow circles) are indicated next to their names. The capital letters indicate various genetic mechanisms of SI. The symbols depicting the *S* genes and *S-like* genes are shown on the bottom.

(Supports Figures 7 and 8).

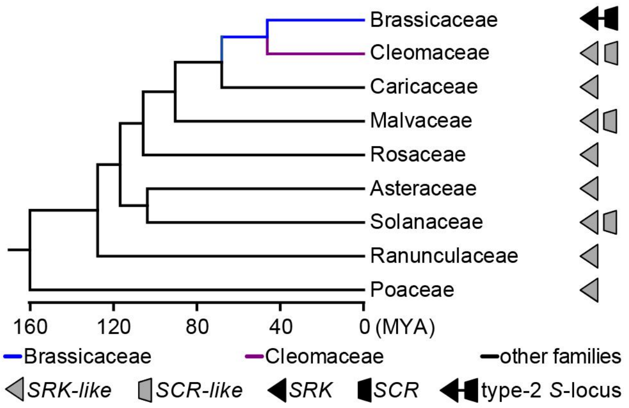

Supplemental Figure S9. The evolution of type-2 SI system in angiosperms.

Distribution of *SRK*/*SRK-like* and *SCR*/*SCR-like* genes in a simplified phylogenetic tree of angiosperm families. Black and gray triangular and trapezoid shapes represent *SRK*/*SRK-like* and *SCR*/*SCR-like*, respectively.

(Supports Figure 7).

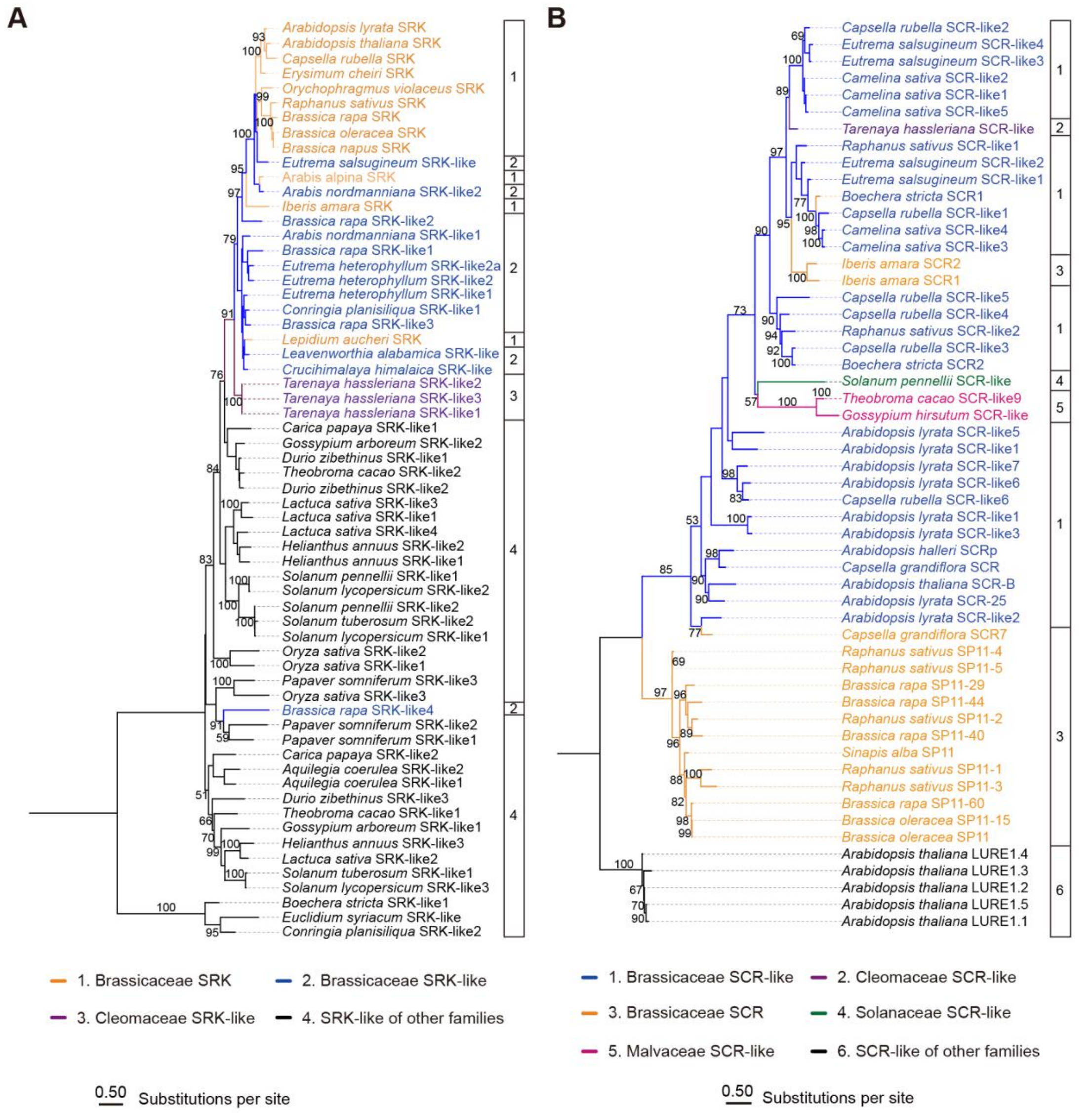

Supplemental Figure S10. Phylogenetic analyses of the *S* genes of type-2 SI.

(A) Maximum-likelihood tree of SRKs/SRK-likes. The phylogenetic tree of SRKs/SRK-likes from 33 monocot and eudicot species, with some bootstrap confidence values above 50% are shown. SRKs/SRK-likes from different evolutionary lineages are indicated by different branch colors.

(B) Maximum-likelihood tree of SCRs/SCR-likes. The phylogenetic tree of SCRs/SCR-likes from 18 species of Brassicaceae, Cleomaceae, Solanaceae and Malvaceae with AtLUREs (*Arabidopsis thaliana* LUREs) as a root, with some bootstrap confidence values above 50% are shown. SCRs/SCR-likes from different evolutionary lineages are indicated by different branch colors.

Please refer to Supplemental File S2 for the detailed bootstrap values.

(Supports Figure 7).

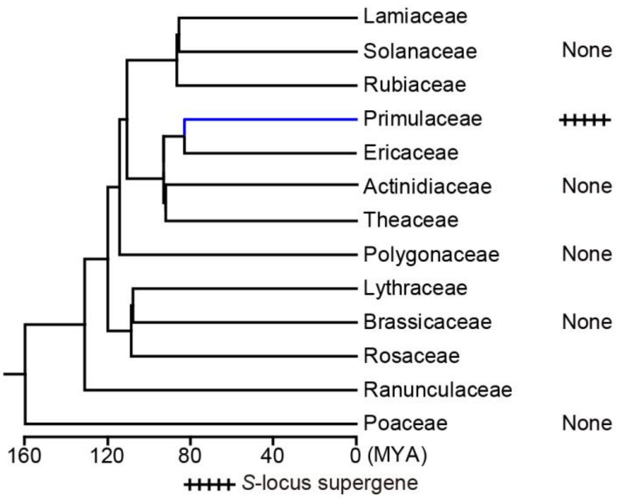

Supplemental Figure S11. The evolution of type-4 SI system in angiosperms.

The origin of the *S*-locus supergene in Primulaceae. A simplified phylogenetic tree of the angiosperm families is shown on the left. The *S*-locus supergene is shown on the right. The comb line represents the *S*-locus supergene; “None” indicates that no *S*-locus supergene-like structures were detected.

(Supports Figure 7).

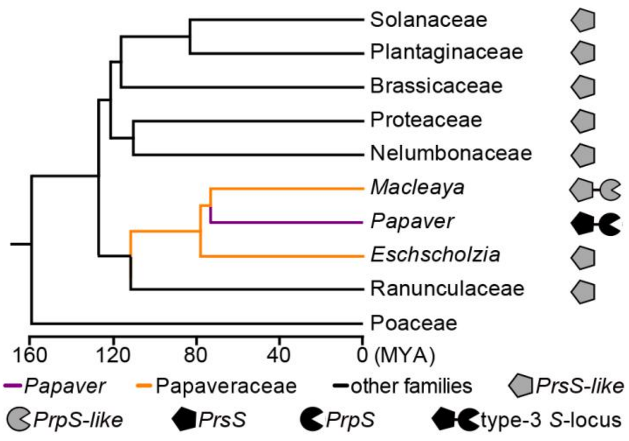

Supplemental Figure S12. The evolution of type-3 SI system in angiosperms.

Distributions of *PrsS*/*PrsS-like* and *PrpS*/*PrpS-like* genes in a simplified phylogenetic tree of the angiosperm families and Papaveraceae subfamilies. Black and gray pentagon and sector shapes represent *PrsS*/*PrsS-like* and *PrpS*/*PrpS-like*, respectively.

(Supports Figure 7).

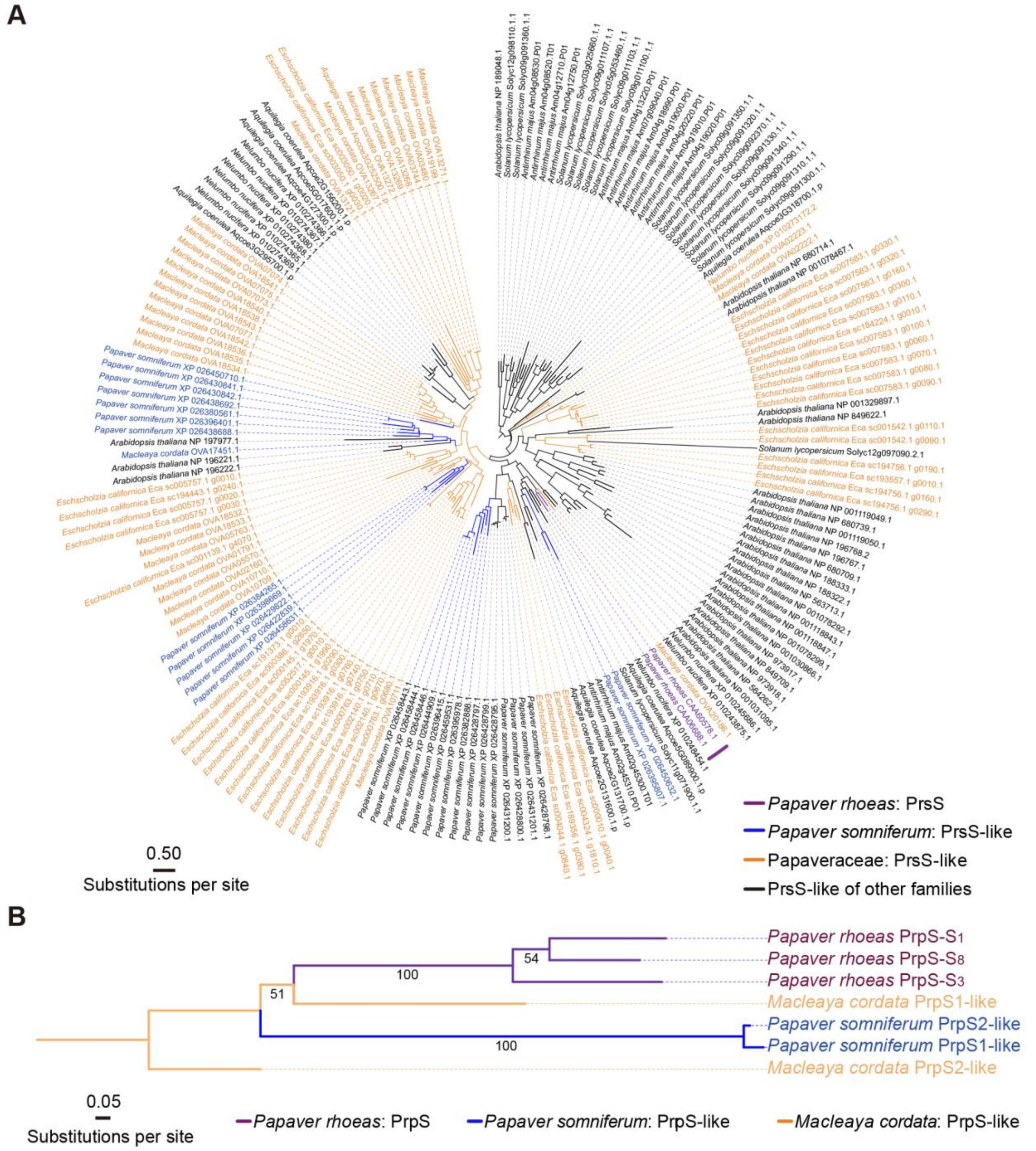

Supplemental Figure S13. Phylogenetic analyses of *S* genes of type-3 SI.

(A) Maximum-likelihood tree of PrsSs/PrsS-likes. The phylogenetic tree of PrsSs/PrsS-likes from 13 eudicot species. PrsSs/PrsS-likes from different evolutionary lineages are indicated by different branch colors. Please refer to Supplemental File S2 for the detailed bootstrap values.

(B) Maximum-likelihood tree of PrpSs/PrpS-likes. The phylogenetic of PrpSs/ PrpS-likes from three Papaveroideae species with bootstrap confidence values above 50% are shown. PrpSs/PrpS-likes from different evolutionary lineages are indicated by different branch colors.

(Supports Figure 7).

Supplemental Table S1. Pollination and genotype analyses of transgenic plants of *ShSLF*s *Ph*S_3S_{3L} (Supports Figure 7).

T_0 plants	Self-pollination[a]	Phenotype	Expected T_1 ratio[b]	Observed T_1 ratio[c]	χ^2 test $\chi^2_{0.01}$=6.64, $\chi^2_{0.05}$=3.84[d]
ShS_5*-SLF5 Ph*S_3S_{3L} #2	5/5	SC	1:1	12:9 (21)	0.22 P>0.05
ShS_5*-SLF5 Ph*S_3S_{3L} #5	7/7	SC	1:1	14:9 (23)	0.54 P>0.05
ShS_5*-SLF6 Ph*S_3S_{3L} #1	4/4	SC	1:1	11:12 (23)	0.02 P>0.05
ShS_5*-SLF6 Ph*S_3S_{3L} #3	5/5	SC	1:1	13:10 (23)	0.19 P>0.05

[a] The number of capsules per number of pollinations.

[b] Expected segregation ratio predicted by competitive interaction with S_3S_{3L}: (S_3S_3 and $S_{3L}S_{3L}$) = 1:1 of T_1 progeny.

[c] Observed segregation ratio of the S_3S_{3L}: (S_3S_3 and $S_{3L}S_{3L}$) of T_1 progeny with their total number in brackets.

[d] The chi-squared goodness-of-fit tests of the observed segregation ratios. $\chi^2_{0.01}$ = 6.64, $\chi^2_{0.05}$ = 3.84. If the χ^2_{sample} < 3.84, the value of P > 0.05, which means the observed segregation ratio is fit to the expected. If 3.84 < χ^2_{sample} < 6.64, 0.01 < P < 0.05, which means there was a significant difference between the observed and expected segregation ratio. If χ^2_{sample} > 6.64, the value of P < 0.01, which means a very significant difference between the observed and expected segregation ratio.

Supplemental Table S2. Pollination and genotype analyses of transgenic plants of *AhSLF*s *PhS_3S_{3L}* and *AmSLFL PhS_3S_{3L}* (Supports Figure 7).

T_0 plants	Self-pollination[a]	Phenotype	Expected T_1 ratio[b]	Observed T_1 ratio[c]	χ^2 test $\chi^2_{0.01}$=6.64, $\chi^2_{0.05}$=3.84[d]
AhSLF-S_1E PhS_3S_{3L} - #1	13/14	SC	1:1	14:15 (29)	0.02 P>0.05
AhSLF-S_1E PhS_3S_{3L} #2	5/5	SC	1:1	31:0 (31)	20.6 P<0.01
AhSLF-S_2C PhS_3S_{3L} #1	7/7	SC	1:1	28:3 (31)	11.9 P<0.01
AhSLF-S_2C PhS_3S_{3L} #2	7/8	SC	1:1	30:1 (31)	17.2 P<0.01
AhSLF-S_4D PhS_3S_{3L} #1	8/13	SC	1:1	21:10 (31)	1.98 P>0.05
AmChr7-SLFL1 PhS_3S_{3L} #1	5/5	SC	1:1	19:12 (31)	0.79 P>0.05
AmChr7-SLFL1 PhS_3S_{3L} #2	5/5	SC	1:1	20:11 (31)	1.31 P>0.05

[a, b, c, d] are identical to Supplemental Table S1.

Supplemental Table S3. Pollination and genotype analyses of transgenic plants of *PaSLFLs* PhS_3S_{3L}, *PaSFBs* PhS_3S_{3L} and *MdSFBB* PhS_3S_{3L} (Supports Figures 4, 5 and 7).

Genotype identification	T_0 plants	Self-pollination[a]	Phenotype	Expected T_1 ratio[b]	Observed T_1 ratio[c]	χ^2 test $\chi^2_{0.01}$=6.64, $\chi^2_{0.05}$=3.84[d]
PCR	*PaS*$_4$*-SLFL1 PhS*$_3$*S*$_{3L}$ #2	4/5	SC	1:1	27:2 (29)	13.4 P<0.01
	PaS$_4$*-SLFL1 PhS*$_3$*S*$_{3L}$ #6	8/10	SC	1:1	22:7 (29)	4.22 P<0.05
	PaS$_4$*-SLFL2 PhS*$_3$*S*$_{3L}$ #1	5/5	SC	1:1	23:6 (29)	5.53 P<0.05
	PaS$_4$*-SLFL2 PhS*$_3$*S*$_{3L}$ #6	5/5	SC	1:1	24:5 (29)	7.06 P<0.01
	PaSFB1 PhS$_3$*S*$_{3L}$ #6	5/5	SC	1:1	19:10 (29)	1.45 P>0.05
	PaSFB1 PhS$_3$*S*$_{3L}$ #10	7/7	SC	1:1	24:5 (29)	7.06 P<0.01
	PaSFB4 PhS$_3$*S*$_{3L}$ #4	5/5	SC	1:1	23:6 (29)	5.53 P<0.05
	PaSFB4 PhS$_3$*S*$_{3L}$ #7	5/5	SC	1:1	28:1 (29)	16.2 P<0.01
	MdSFBBβ-S$_9$ *PhS*$_3$*S*$_{3L}$ #7	7/10	SC	1:1	14:3 (17)	4.81 P<0.05
	MdSFBBβ-S$_9$ *PhS*$_3$*S*$_{3L}$ #9	7/8	SC	1:1	14:1 (15)	8.20 P<0.01
Southern blot	*PaS*$_4$*-SLFL1 PhS*$_3$*S*$_{3L}$ #4	10/10	SC	1:1	20:6 (26)	4.06 P<0.05
	PaS$_4$*-SLFL2 PhS*$_3$*S*$_{3L}$ #7	10/10	SC	1:1	21:5 (26)	5.44 P<0.05
	PaSFB1 PhS$_3$*S*$_{3L}$ #5	6/9	SC	1:1	19:7 (26)	2.93 P>0.05
	PaSFB4 PhS$_3$*S*$_{3L}$ #2	3/5	SC	1:1	23:3 (26)	9.03 P<0.01

[a, b, c, d] are identical to Supplemental Table S1.

Supplemental Table S4. Pollination and genotype analyses of transgenic plants of *AcSLF*s *PhS_3S_{3L}* (Supports Figures 6 and 7).

T_0 plants	Self-pollination[a]	Phenotype	Expected T_1 ratio[b]	Observed T_1 ratio[c]	χ^2 test $\chi^2_{0.01}$=6.64, $\chi^2_{0.05}$=3.84[d]
AcSC4-SLF4 PhS_3S_{3L} #3	6/8	SC	1:1	16:4 (20)	3.96 $P<0.05$
AcSC4-SLF4 PhS_3S_{3L} #5	5/5	SC	1:1	13:3 (16)	3.46 $P>0.05$
AcSC4-SLF5 PhS_3S_{3L} #3	7/7	SC	1:1	13:6 (19)	1.37 $P>0.05$
AcSC4-SLF5 PhS_3S_{3L} #4	8/10	SC	1:1	10:6 (16)	0.56 $P>0.05$

[a, b, c, d] are identical to Supplemental Table S1.
